# The Interplay Between Poor Sleep and Work-Related Health

**DOI:** 10.3389/fpubh.2022.866750

**Published:** 2022-07-07

**Authors:** Ingo Fietze, Lisa Rosenblum, Matthew Salanitro, Alexey Danilovich Ibatov, Marina Vladimirovna Eliseeva, Thomas Penzel, Désirée Brand, Gerhard Westermayer

**Affiliations:** ^1^Interdisciplinary Sleep Medicine Center, Charité—Universitätsmedizin Berlin, Berlin, Germany; ^2^The Federal State Autonomous Educational Institution of Higher Education I.M. Sechenov First Moscow State Medical University of the Ministry of Health of the Russian Federation, Moscow, Russia; ^3^Department of Neurophysics, Philipps-Universität Marburg, Marburg, Germany; ^4^BGF Gesellschaft für Betriebliche Gesundheitsförderung mbH, Berlin, Germany

**Keywords:** health hazard, occupational health management, insomnia, sleep disorder, work condition

## Abstract

**Objectives:**

Sleep disorders can arise from work. Employees who experience work overload are more likely to develop sleep problems. Poor sleep leads to decreased performance, sick leave, and accidents. Therefore, sleep disorders may be linked to workplace hazards as well as decreased occupational health, however, the relationship remains unknown.

**Methods:**

This relationship was examined using secondary data analysis of aggregated survey data from 97 companies based in Germany between 2003 and 2020 as part of Workplace Health Management project. Two extreme groups with respect to sleep problems were analyzed (*N* = 4,865 + 9,795). The survey “Diagnosis of corporate health” contained 137 individual questions which recorded all relevant working conditions, aspects of health, and one question relating to insomnia traits. A one-way analysis of variance was used to examine whether and to what extent the potentials, hazards, and health aspects differed between employees depending on their perceived sleep problems. In addition, multiple linear regressions were used to determine whether and to what extent work characteristics affect various health aspects for both good and poor sleepers.

**Results:**

In total, 49.7% of staff reported moderate difficulty falling and/or remaining asleep. These poor sleepers perceived all health potentials worse than good sleepers, especially on scales such as fair assessment, work climate, and learning at work. Furthermore, poor sleepers perceived health hazards (physical environmental stress, job insecurity, and time pressure) more whilst positive health indicators (joy of work and confidence) were perceived less.

**Conclusion:**

Overall, the determination of sleep difficulties could be used as a substantial health indicator. Also, these sleep problems are reported more frequently in certain occupations compared to others, which could mean that the perception of sleep health varies between professions. Therefore, it is important to implement specific recommendations for each industry in order to improve working conditions for poor sleepers which in turn, improves their health.

## Introduction

In the context of company health management, prophylactic measures are becoming increasingly important when trying to prevent sleep-wake difficulties. Strenuous demands such as shift work and Covid-19 management can promote these difficulties by negatively affecting the mental and physical health of employees (1–3). This means that work-related sleep disorders are becoming more prevalent and the consequences that come with them should be addressed. For instance, insufficient sleep has been linked to slower information processing, impaired cognition, and restricted task performance (4). Consequently leading to presenteeism, this is when an employee's productivity is diminished due to limited cognitive functioning (5). Poor sleep can also be a risk factor for illnesses such as diabetes, hypertension, and depression (6). As a result, these illnesses may give rise to absenteeism which is when an employee suffers from long-term sickness and takes many unplanned absences (7). Therefore, poor sleep could moderate health and cognitive functioning of an employee (8).

Until now, company health management has primarily focused on sleep problems in shift work—under which women suffer from more than men regarding to their sleep health (9). Research from Theorell et al. (10) stated the initial approaches toward finding interrelationships between job strain and sleep disorders: they demonstrated that higher work strain with a low level of decision-making leeway can increase the prevalence of sleep disorders. In this context, excessive work strain entails a combination of great psychic stress and a lack of self-control (11). These and further investigations led to the following scientifically supported conclusion by the Swedish Council for the Assessment of Health Technologies (12): company staff with perceived work overload are more likely to develop sleep disorders. In addition, excessive job demands, shift work, and bullying at work may exacerbate these sleep disorders. Research from 2012 reveals that 13–22% of employees suffer from excessive workload which could mean that there is a great risk of developing sleep disorders in the working population (13).

In our initial study, out of the 372 shift workers from the region of Berlin-Brandenburg in Germany, 46% reported complaints of excessive fatigue, and 30% had difficulty falling asleep and/or maintaining sleep (14). We also found a negative evaluation of subjectively perceived work conditions such as enjoyment of work, irritability, exhaustion, and physical impairment. For 79 of these shift workers, we also applied actigraphy to study their sleep for 14 days. This disclosed that poor sleep efficiency was associated with greater levels of exhaustion during work. Similarly, research from Littwiller et al. (15) demonstrated the negative relationship between sleep duration and sleep quality with workload, exhaustion, and depression.

These health-relevant and work-related studies indicate that there is a need for data collection within the context of company health management and from the standpoint of sleep research. Especially if we are aiming to support employers in making effective operational adjustments toward enhancing sleep health. In terms of cost factors, recent research has shown that a poor sleeper annually costs their employer 2,280 dollars more per year, both as a result of the ineffective presence and absenteeism (8). Therefore, if sleep health adjustments are implemented in the workplace it may benefit both the employee and the employer.

For many years now, the company *BGF GmbH*, Berlin, Germany (BGF) has conducted standard surveys in companies, institutions, and industrial groups on the topics of health potentials, health hazards, and health indicators [Questionnaire on the Diagnosis of Corporate Health: *Fragebogen zur Diagnose Betriebliche Gesundheit*, Westermayer and Stein (16)]. This contains questions relating to health indicators of psychological impairments and it also includes one sleep-related question which is “I often have sleep problems (trouble falling and remaining asleep).” This question relates to the most frequent sleep disorder, insomnia which has a significant effect on the performance and the mood of the employee on the following day (17).

The present study aims to analyze the impact of subjectively perceived difficulties in falling asleep and/or maintaining sleep on a person's working life. The goal of this study is to investigate which work-related health potentials and health hazards are closely linked to subjectively reported sleep problems.

## Materials and Methods

### The Survey

The present study is a secondary data analysis of aggregated survey datasets collected from 2003 to 2020 as part of Workplace Health Management project. In total, 97 companies in Germany took part (mainly in Berlin, Brandenburg, and Mecklenburg-Western Pomerania), in each case they used the above-mentioned instrument for the diagnosis of corporate health. This instrument was developed and tested by Ducki (18, 19) on behalf of BGF and was found to be satisfactory. Over the years the constructs covered have been constantly revised, and new topics have also been added—either at the request of companies and/or because certain topics have become more important. The one question relating to insomnia traits remained unchanged throughout the whole study. The current version, which was also used here, was tested again by Brand (20) with regard to reliability (internal consistency with Cronbach's alpha and coefficients of selectivity) and was also shown here to be satisfactory (Supplementary Table 1).

The instrument uses 137 individual questions to measure a wide range of working conditions and aspects of health. It also contains one question relating to insomnia. All items could be answered with a Likert scale ranging from 1 (completely agree) to 5 (strongly disagree) which were recoded into 0% (strongly disagree) to 100% (strongly agree) in order to improve the comprehensibility of the results for each company. These 137 individual questions were split into 24 scales, with the scale mean being formed from the item means in each case. The mean of each of these scales depicts the average agreement on that specific scale. There were four scales for health indicators that captured the aspects of health: these were surveyed with a total of 35 items. There were 13 scales for potentials: specifically, factors potentially conducive to health which consisted of 65 items. Lastly, there were seven scales for hazards potentially detrimental to health: these consisted of 37 items. A description of all scales including the quality test can be found in Supplementary Table 1.

**Figure 1:**
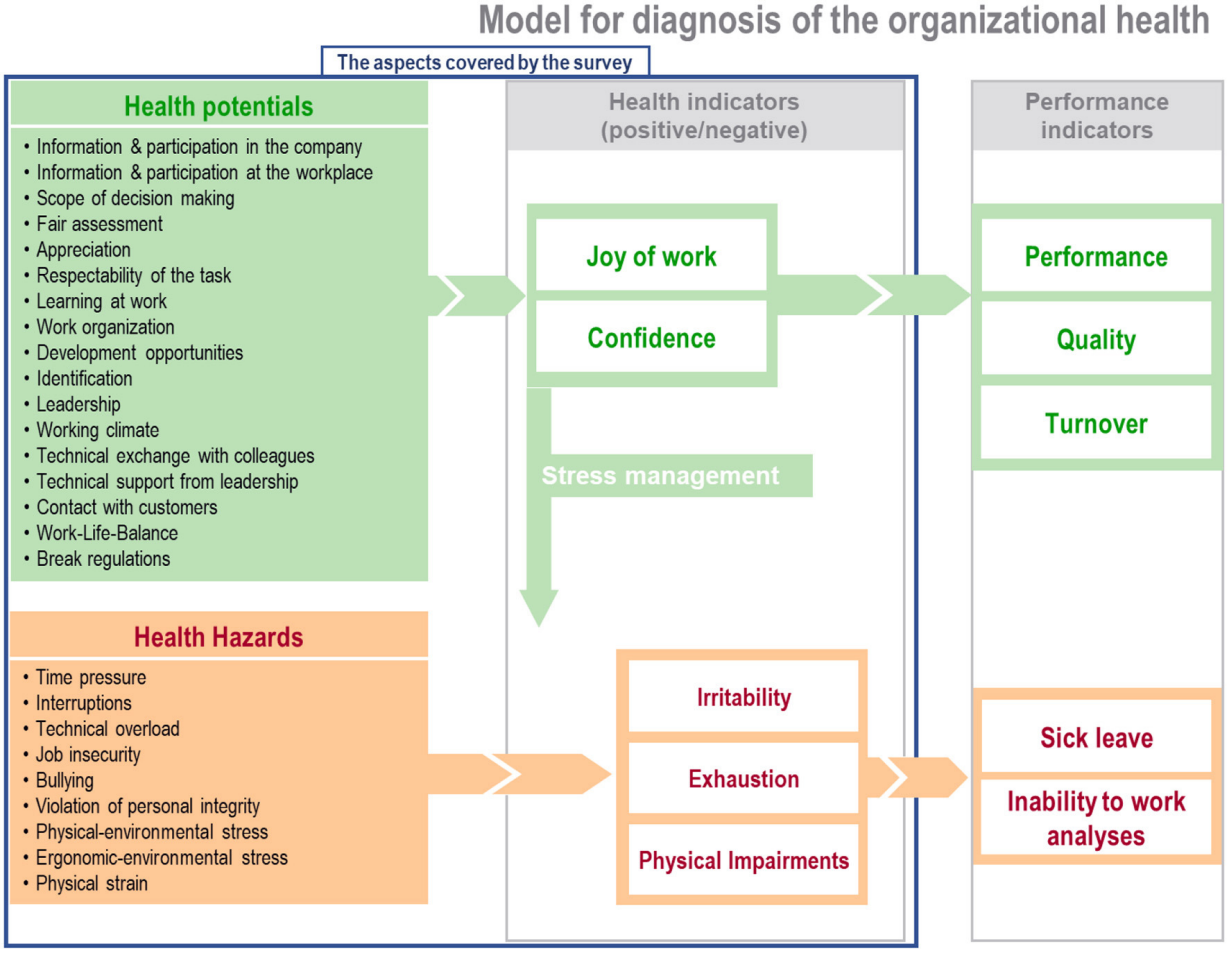
Model for the diagnosis of corporate design (^©^BGF GmbH).

Figure 1 elaborates on the areas of content being surveyed, as well as the influencing interrelationships. Health potentials—to the extent they are perceived—exert positive effects on our well-being, while health hazards negatively affect our well-being. Positive health indicators are joy of work and confidence [following the sense of coherence (SOC) elaborated by Antonovsky (21)], negative health indicators are psychological and physical impairments that—depending on intensity and duration—can lead to illness or sick leave. The scope of questions covers the left and the central columns in Figure 1 and the performance indicators are shown in the right column.

The following socio-demographic information about the employee was also recorded in the survey: age (in age groups), gender (male/female), working hours (full-time/part-time), and management function (yes/no). Personal data such as name, date of birth, and address were not collected which meant that there were no conflicts of ethical or data protection. The year of the survey, the place of the survey, and the economic sector of the company were also added as variables in the aggregated data set.

### Description of the (Typical) Procedure When Initializing a Survey in a Company

The employees were informed of the survey by organized informative events or internal company circulars. On average 60% of the employees from each company responded to the survey. Taking part was voluntary, and the data collected was anonymous—surveys were completed either on paper forms or via an online platform. There was no individual monetary compensation for the time and effort spent in completing the questionnaires, however filling out the survey took place during working hours for all cases. The survey projects were in all cases approved by the employees' respective workers' council and staff council. Distribution of the questionnaires took place through in-house channels by the following: company health managers, company managers, and similar personnel. In some cases, the link to the online platform was provided.

### Aim

The present study aimed to see whether a relationship existed between subjectively evaluated sleep quality and perception of health potentials, health indicators, and health hazards.

### Data Processing

In order to investigate possible differences in the perception of working conditions and health between respondents with and without sleep problems, two extreme groups were formed: Respondents who answered the question “I often have sleep problems (difficulty falling asleep, difficulty staying asleep).” “completely” or “mostly agree” (*N* = 4,865) were combined into the group “moderate/severe sleep problems” (poor sleepers), while those who indicated “little” or “not at all” (*N* = 9,795) were combined into the group “little/no sleep problems” (good sleepers). All further calculations refer to these two groups. Two tables were created to describe the sample. For the first table, the above-mentioned sleep question was distributed according to age and gender. For the second table, the above mentioned extreme groups were broken down by industry and gender. To provide clear and coherent results we excluded the respondents who answered “partly agree” in the sleep question from all analyses as this was a midpoint. Respondents in this group could lean toward either group which means if we combine these to one of the extreme groups we could create bias.

### Statistical Analyses

All analyses were carried out with the statistics program SPSS, Version 27. A one-factor analysis of variance (ANOVA) was performed to test for significant differences between good sleepers and poor sleepers for all scales recorded (health indicators, potentials, and hazards). The next step was to determine whether and to what extent the perception of health of the two groups was influenced by working conditions. For this purpose, a multiple linear regression was conducted for each health indicator, this entailed using all recorded working conditions as independent variables. This was the crude model (Model 1) which initially ignored covariates. Then it was adjusted for the confounding variables age, gender, and industry (Model 2 and 3). Finally, the four health indicators (joy of work, confidence, psychological and physical impairments) were combined in a health index and a multiple linear regression was conducted.

The datasets used in this research were not completely identical for the following reasons: They were collected over decades, adapted to internal company circumstances, expanded to include current topics/problems, and not enough demographic data. This was counteracted in the present analysis by not including scales or topic areas (working conditions) that were recorded in isolated cases in the final analysis. This meant that from the initial 126 individual questions regarding 26 working conditions, 102 questions regarding 20 working conditions remain. However, there are still a few missing values—either because a scale was not recorded in a single company or due to individuals missing a question. See Supplementary Table 5 for the total sample per scale.

## Results

At the time of this analysis, the aggregated dataset contained 19,852 questionnaires. In 19,504 of the questionnaires, the participants provided their answer to the question “I often have sleep problems (trouble falling and/or staying asleep).” The gender of the respondents was 52.9% women and 33% men; 14.1% of those responding did not indicate their gender. The survey results revealed that half of the respondents suffered either from only slight (22.8%) or no (27.4%) sleep problems. Additionally, 24.8% responded that they partly suffered from sleep problems, 13.1% mostly suffered, and 11.8% permanently suffered (Supplementary Table 2). Consequently, around 50% suffer from a sleep problem, and 25% of these suffer from moderate/severe sleeping problems.

When we consider these sleep problems broken down by age and gender, females in the 50 or older age group more frequently self-reported moderate to severe sleeping problems (33.2 %) compared to all other age groups (Table 1). Therefore, perceived sleep problems may be more prevalent in females when they reach 50 years of age or older. In contrast, this was not seen among male employees. The frequency of self-reported moderate/severe sleeping problems was similar across age groups. When we break these sleep problems down by industry and gender the results indicate that sleep problems are more frequently self-reported in the manufacturing industry, health care industry, and the economic service companies than in any other industry for both men and women. Therefore, employees working in these industries may be more susceptible to developing sleep problems (Supplementary Table 3).

**Table 1:** A table to show the frequency of respondents to the sleep question, broken down by age and gender.

**Age group**	**Moderate/severe sleep problems**		**Little/no sleep problems**	
	***N***	**%**	***N***	**%**
Male	1,375	21.4	3,552	55.2
Up to 29 years	240	23.1	596	57.4
30–39 years	303	21.2	793	55.3
40–49 years	365	20.1	936	53.7
50 years and older	408	21.6	1,024	54.1
N.a.	59	17.9	203	61.7
Female	2,841	27.6	4,787	46.4
Up to 29 years	350	26.1	680	50.7
30–39 years	437	21.9	1,077	53.9
40–49 years	826	26.6	1,448	46.6
50 years and older	1,081	33.2	1,270	39
N.a.	147	24.2	312	51.4
N.a.	649	23.5	1,456	52.8
Up to 29 years	52	22.3	142	60.9
30–39 years	93	23.1	216	53.7
40–49 years	128	20.8	339	55
50 years and older	135	25.9	254	48.7
N.a.	241	24.5	505	51.3
Sample in total	4,865	24.9	9,795	50.2
Up to 29 years	642	24.6	1,481	54.3
30–39 years	833	21.7	2,086	54.4
40–49 years	1,319	24.1	2,723	49.8
50 years and older	1,624	28.6	2,548	45
N.a.	447	23.3	1,020	53.1

**Figure 2:**
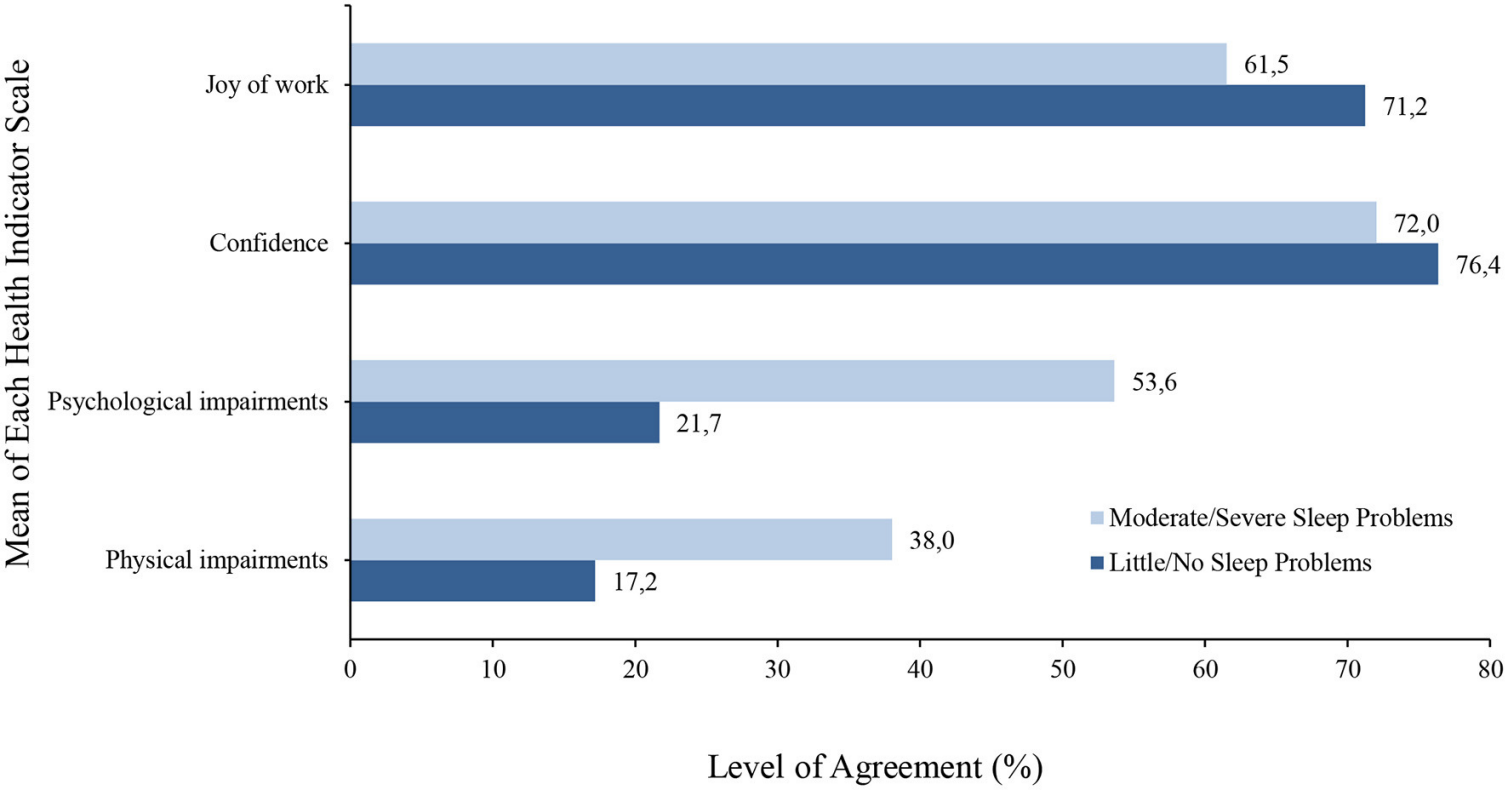
A bar graph to show the mean level of agreement for each health indicator (scales that refer to positive health indicators are joy of work and confidence, negative health indicators are psychological and physical impairments that—depending on intensity and duration—can lead to illness or sick leave) in both good and poor sleepers.

**Figure 3:**
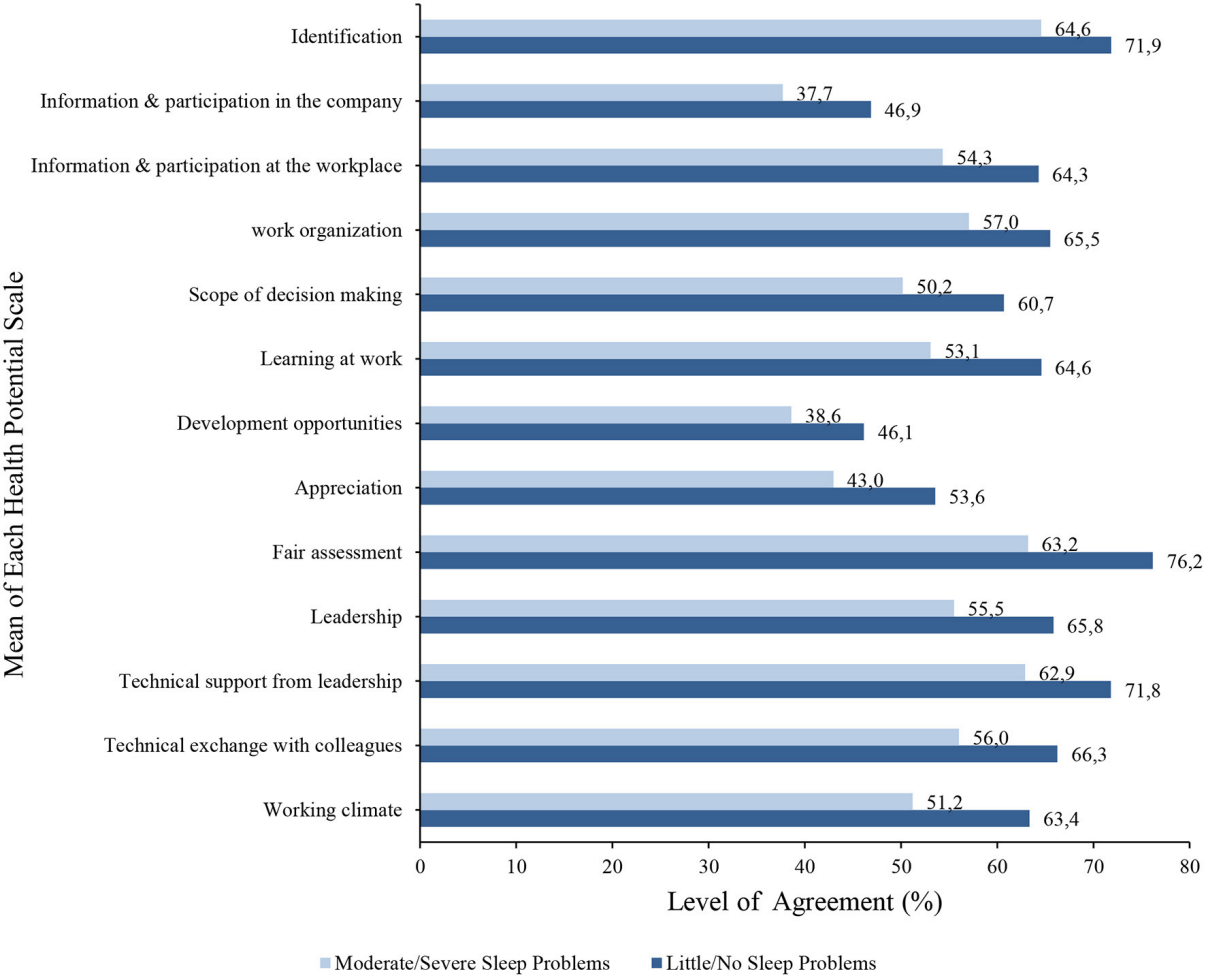
A bar graph to show the mean level of agreement for each health potential (scales that refer to the positive effects on our well-being) in both good and poor sleepers.

**Figure 4:**
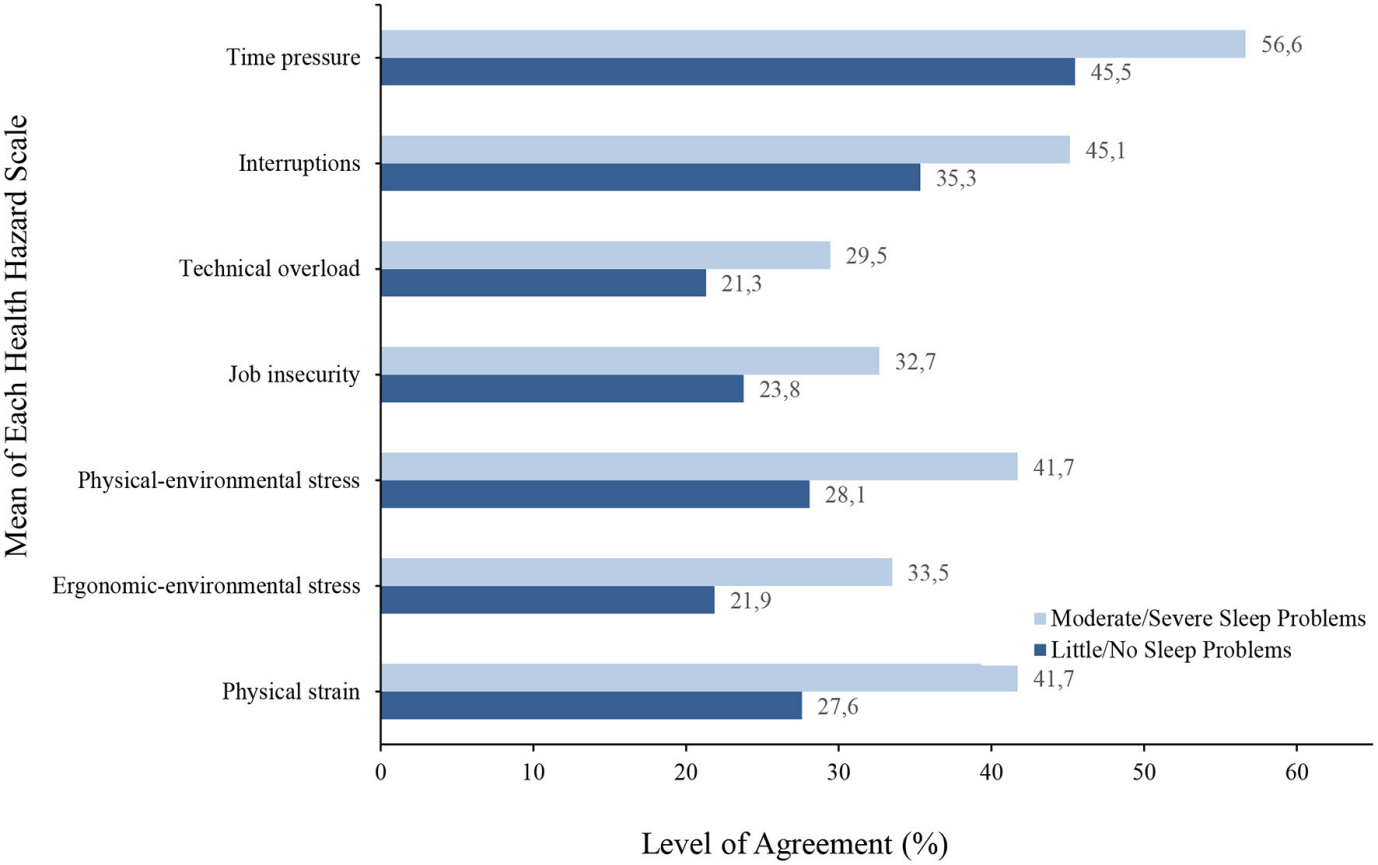
A bar graph to show the mean level of agreement for each health hazard (scales that refer to the negative effects on our well-being) in both good and poor sleepers.

The one-factor ANOVA concerning the two extreme groups “little/no sleep problems” and “moderate/severe sleep problems" showed significant differences for all scales recorded (health indicators, health potentials, health hazards) (Supplementary Table 4). See Supplementary Table 5 for the means and standard deviations of each health aspect scale for both groups. Particularly large differences (more than 10% difference each) were found in the health indicators for perceived psychological and physical impairments (Figure 2). Here, poor sleepers were shown to be more than twice as impaired (53.6 vs. 21.7% and 38 vs. 17.2%) than good sleepers. Poor sleepers perceived all health potentials worse especially on scales such as fair assessment (decrease 13%), work climate (12.2%), and learning at work (11.5) compared to good sleepers (Figure 3). In contrast to the health potentials, the health hazards are weighted more by poor sleepers. They reported more physical strain (increase 14.1%), physical environmental stress (13.6%), ergonomic environmental stress (11.6%), and time pressure (11.1%) compared to good sleepers (Figure 4).

See Supplementary Tables 6, 7 for the tables concerning multiple linear regressions with the health index (sum score of the joy of work, confidence, psychological, and physical impairments) as the outcome and aspects of health (work-related potentials and hazards) as independent variables. These analyses were differentiated into good sleepers and poor sleepers. The following aspects of health which are related to good work health were shown to have a strong relationship with good sleepers and slightly weaker in poor sleepers: Technical overload had a more negative relationship with the health of good sleepers *N* = 4,659, ß = −0.541, 95% CI[−0.603, −0.479], *p* < 0.001, R^2^ = 44.0% whilst poor sleepers had a weaker negative relationship *N* = 2,456, ß = −0.525, 95% CI[−0.616, −0.434], *p* < 0.001, R^2^ = 39.4% in the adjusted model. Identification with work had a positive relationship with the health index for good sleepers *N* = 4,659, ß = 0.480, 95% CI[0.419, 0.542], *p* < 0.001, R^2^ = 44.0%, whilst poor sleepers had a slightly weaker positive relationship *N* = 4,659, ß = 0.463, 95% CI[0.373, 0.554], *p* < 0.001, *R*^2^ = 39.4% in the adjusted model. Furthermore, learning at work had a positive relationship with the health index for good sleepers *N* = 2,456, ß = 0.483, 95% CI[0.429, 0.536], *p* < 0.001, R^2^ = 44.0%, whilst poor sleepers had a slightly weaker positive relationship *N* = 2456, ß = 0.396, 95% CI[0.313, 0.479], *p* < 0.001, *R*^2^ = 39.4% in the adjusted model. In terms of poor work health, physical environmental stress and job insecurity had a negative relationship with the health index for poor sleepers (*N* = 2,456, ß = −0.272, 95% CI[−0.364, −0.180], *p* < 0.001, *R*^2^ = 39.4% and *N* = 2,456, ß = −0.181, 95% CI[−0.250, −0.112], *p* < 0.001, *R*^2^ = 39.4%), respectively. Whilst good sleepers showed a slightly weaker negative relationship (*N* = 4,659, ß = −0.178, 95% CI[−0.240, −0.117], *p* < 0.001, *R*^2^ = 44.0% and *N* = 4659, ß = −0.122, 95% CI[−0.170, −0.070], *p* < 0.001, *R*^2^ = 44.0%), respectively.

The following positive work attributes were shown to have a positive relationship with the health index for poor sleepers and a slightly weaker positive relationship for good sleepers in the adjusted model: scope of decision making for poor sleepers *N* = 2,456, ß = 0.237, 95% CI[0.161, 0.314], *p* < 0.001, *R*^2^ = 39.4% and for good sleepers *N* = 4,659, ß = 0.110, 95% CI[0.061, 0.159], *p* < 0.001, *R*^2^ = 44.0%). Work organization for poor sleepers (*N* = 2,456, ß = 0.138, 95% CI[0.011, 0.264], *p* < 0.001, *R*^2^ = 39.4% and for good sleepers *N* = 4,659, ß = 0.111, 95% CI[0.030, 0.192], *p* < 0.001, *R*^2^ = 44.0%). Technical exchange with colleagues for poor sleepers (*N* = 2,456, ß = 0.139, 95% CI[0.060, 0.218], *p* < 0.001, *R*^2^ = 39.4% and for good sleepers *N* = 4,659, ß = 0.098, 95% CI[0.048, 0.149], *p* < 0.001, *R*^2^ = 44.0%). Additionally, see Supplementary Tables 8–15 for output tables for the multiple linear regressions regarding the relationship between the individual health indicators with each health attribute, in both good and poor sleepers.

## Discussion

Our study has demonstrated that sleep problems—specifically difficulties in falling asleep and/or maintaining sleep—occur in many employees from different disciplines. This suggests that sleep problems are a relevant issue in the everyday working world. Additionally, the prevalence of sleep problems seems to be more pronounced in certain industries than others. Furthermore, work-related health potentials, health indicators, and health hazards were perceived differently by good sleepers in comparison to poor sleepers. These findings provide further insights and opportunities for workplace health promotion.

Approximately 50% of all those surveyed suffer from moderate to severe symptoms of insomnia. Women more frequently reveal these traits, and the prevalence seems to largely increase with older age. Our results thereby concur with findings from the literature (22–24). Regarding industry, the highest frequency of self-reported sleep problems came from employees in the service, health, and manufacturing industry. The reason for health and manufacturing industries may be due to irregular work schedules such as shift work. Unfortunately, we did not ask about shift work, however, it is well known from the literature that shift work is a risk factor for disturbing a normal sleeping pattern (25).

Differences were found in health indicators, health potentials, and health hazards for poor and good sleepers. For instance, good sleepers experienced more work joy and confidence than poor sleepers. Also, their response to all health potential scales related to joy and confidence showed higher levels of agreement toward positive work health than those of poor sleepers. In contrast, poor sleepers reported more physical and mental impairments and their response to all health hazard scales showed higher levels of agreement toward poor work health than those of good sleepers. This indicates that there may be a negative relationship between poor sleep and perception of negative health aspects from work. This relationship could also work the opposite way. For instance, time pressure on tasks, job security, and stress could play a pivotal role in employees' sleep or vice versa. This reveals entirely new standpoints concerning recommendations for action by employers.

The results from the multiple linear regression showed that the health of good sleepers benefitted more from the improvement of soft facts such as identification and learning at work. Whilst the health of poor sleepers benefitted more from the improvement of hard facts such as physical-environmental stress, job insecurity, and good work organization. This means that when improving working conditions, sleep health should be investigated in advance to be able to provide employees with successful individual health initiatives. Our study, therefore, complements previous work in this area very well:

Research from Magnavita and Garbarino (26) investigated the issues of sleep, health, and wellness at work—including sleep deficits, non-restful sleep, sleep disturbances, and faulty perception of sleep—with particular consideration to studies involving employers. Their findings revealed that some professional factors can negatively influence sleep such as shift work, isolation, violence at the workplace, psychosocial stress, gender relevance, cultural factors, and behavioral factors. In addition to this, research from Schierholz et al. (27) reported that 84 medical staff members demonstrated sleep impairments influenced by professional anxiety and constant on-call work conditions. In our current study, we showed similar findings for poor sleepers, they self-reported more negative health hazards from work and a high frequency of poor sleepers came from the medical field.

Furthermore, the relationship between workload and poor sleep was also reported by Leitaru et al. (28). They showed that sleep is moderated by many factors for instance, bullying at the workplace strengthens this relationship (29), and physical activity diminishes it (28). This suggest that poor sleep—especially sleep disorders—can moderate well-being, health, and productivity. It can accordingly configure a vicious circle or can represent a bidirectional relationship between productivity and health of the employees. Halonen et al. (30) reported that increasing workload can provoke or trigger insomnia and that a reduction in workload can decrease the incidents of insomnia. This study investigated employees (88% female) in middle age (mean age 44 years) for 12 years. Not only was the workload a distinct risk factor for insomnia it was also found to be a primary cause of burnout, but not without consideration of poor sleep. Metlaine et al.‘s (31) study of 1,300 employees in the financial sector demonstrated that burnout particularly occurs when insomnia has developed. This emphasizes the significance of how insomnia can be both the consequence and the cause of negative work conditions. In addition to this, our study also showed that the health hazards including time pressure, technical overload, and physical strain—which are all related to work overload—are perceived much more by poor sleepers than by good sleepers. Therefore, this current research may provide further evidence in support of those previous findings.

One benefit of the cited work and the current study is that the analyses provide concrete areas of action for the employer: a job-specific guide so to speak, for employees and health experts. Until now, there have primarily been recommendations for shift workers which include aid in preventing shift-workers syndrome and for employers regarding the organization of shift work (25). These recommendations were highlighted in our previous survey on sleep and shift work and it became apparent that permanent night shifts are associated with less irritability, exhaustion, and physical impairment (14). However, there is also evidence of exacerbated health hazards that are especially associated with permanent night shifts (32). More recently, recommendations include self-supervision and collaboration in time scheduling. These were applied to female nurses which resulted in an increase in positive work attitudes (33). Kecklund et al. (34) came to a similar conclusion with police workers: it was considered optimal to allow at least 16 h of off-duty when moving from one shift to an earlier one, even when this meant fewer full days off. On the other hand, they recommend that free choice of shifts is a better solution for work-schedule optimization, especially from ergonomic and health standpoints. In addition to work-schedule optimization (35), physical environmental conditions such as lighting, noise, and posture (standing or sitting) at the workplace can influence health hazards like physical and ergonomic environmental stress. Knowing this and with the new insights from our current survey, it is possible for employers to make industry-specific changes as well as physical environmental changes to improve the sleep health of employees.

In the present study, we compared four health indicators (joy of work, confidence, psychological and physical impairments) with an existing and subjectively perceived sleep problem (insomnia traits). These traits seem to be more strongly associated with psychological and physical impairments than job satisfaction and confidence. Furthermore, work-related health potentials are less strongly associated with poor sleepers compared to health hazards. This suggests that a change of guidelines for promoting workplace health is needed such as individual recommendations according to age, gender, physical and mental fitness, and health status (including existing sleep problems). Our survey precisely makes these recommendations for possible actions, especially if the interrelationships are broken down into individual questions. For example, professional overload and learning at work have a significant influence on health in both good and poor sleepers. In this case, it is recommended to provide sufficient preparation for work tasks and to emphasize the development of skills and abilities of employees. If an employer recognizes or already knows these interrelationships, it is possible to provide much more targeted and effective health management, including sleep health.

There are already several programs for the enhancement of sleep-waking management (36–38). One to mention is to implement prophylactic health measures specifically for the workplace such as fitness exercises for sedentary work and sleep-waking tips for sleep disorders. Workplace-specific prophylactic healthcare can take place face-to-face or, increasingly now, digitally. Although the available data here is still insufficient, initial studies have shown that such digital health interventions can have a positive effect on sleep, organization of staff break routines, mental health, and work satisfaction. A crucial prerequisite here is that staff can directly download the healthcare programs directly to their computers and execute them at their workplaces (36). Another method to enhance sleep-waking management is to implement changes in work conditions (39). This uses similar measures recommended by the Swedish Council for the Evaluation of Health Technologies (12) which include social support during work, large contributions on work-related decisions, and fairness at the workplace. In this current research, we also include scales on fair assessment, participation at the workplace, appreciation, and much more. Therefore, our study provides support to previous literature and emphasizes the recommendations for the improvement of sleep-waking management.

One strength of our study is the large sample size and the number of different branches involved in the research. A large sample size provides a precise approximation of the true beliefs of an average employee concerning sleep and workplace health across multiple disciplines. In contrast, it could be argued that this was a convenience sample due to the responses of the employees being on a voluntary basis. This could increase the likelihood of adding a bias toward our results by having an unrepresentative sample—those who feel more psychological and physical strain or who were bored. Therefore, this sample may potentially overestimate their actual health-related work problems and ignore those who had no time to answer this questionnaire.

In addition, the perception of working conditions and attitudes may have changed significantly since the beginning of this data collection as it was collected over several decades. Especially since the Covid-19 pandemic started, working from home occurs more frequently (40). This could drastically change the opinion of an employee on their occupational health. Unfortunately this alongside other potential confounding variables such as year of survey completion were not investigated in this study. It would be useful to investigate this in future research.

Another limitation of our study is that we used a single question to identify the two most frequent traits of insomnia: difficulties in falling asleep and/or maintaining sleep. Furthermore, we did not collect quantification of the problems (e.g., how many times a week the problems occur) and we did not cover excessively early morning awakening, nor other frequent sleep disorders such as the restless-legs syndrome or sleep-related breathing disorders. Also, we did not collect data objectively (actigraphy or polysomnography). Since insufficient sleep is the most common sleep problem regardless of age and, unlike sleep apnoea or restless legs syndrome, almost always results in nonrestorative sleep, our question has mapped at least one of the most important sleep medical problems.

Another point to consider is that our data lacked person-specific demographics such as body size and weight, comorbidity, consumption of medication or drugs, and family status. Insufficient social support at home can have an additional influence on workload. If, for example, the joy of work can be explained to a level of 42.2% by contact with customers, learning at work, identification, and respectability of one's work, then this leaves 57.8% that may be influenced by social conditions such as family burdens, financial worries and similar. Olson et al. (41) reported in 2015 that an improvement of work-family conflicts can have positive effects on sleep. However, our data suggest that diverse work conditions and work atmospheres may contribute to possible sleep problems, especially for certain age and gender groups. Consequently, it could negatively impact a company's economic viability (Figure 1).

In sum, our results provide new insights into the interrelationship between workload and work conditions. This is important as they both can affect sleep health and can influence the perception of work-related health potentials and hazards. One of the main benefits of using this survey to identify sleep problems in employees is that it provides employers with new ideas and areas of improvement for specific company health management. For instance, modification of work conditions and work environment, as well as workload with a focus toward enhanced sleep.

## Data Availability Statement

The data analyzed in this study is subject to the following licenses/restrictions: Data owned by Gesellschaft für Betriebliche Gesundheitsförderung mbH, Berlin, Germany (BGF). Requests to access these datasets should be directed to info@bgf-berlin.de.

## Ethics Statement

Ethical review and approval was not required for the study on human participants in accordance with the local legislation and institutional requirements. The patients/participants provided their written informed consent to participate in this study.

## Author Contributions

IF: writing, planning, and management. LR: analysis and writing the results. MS: writing, formatting, tables, and figures. AI and ME: planning, statistics, and discussion. TP: planning and management. DB and GW: calculations, tables, figures, and writing the methods. All authors are responsible for the content and writing of the paper. All authors contributed to the article and approved the submitted version.

## Conflict of Interest

DB was employed by BGF Gesellschaft für Betriebliche Gesundheitsförderung mbH. The remaining authors declare that the research was conducted in the absence of any commercial or financial relationships that could be construed as a potential conflict of interest.

## Publisher's Note

All claims expressed in this article are solely those of the authors and do not necessarily represent those of their affiliated organizations, or those of the publisher, the editors and the reviewers. Any product that may be evaluated in this article, or claim that may be made by its manufacturer, is not guaranteed or endorsed by the publisher.

## References

[1] Kronholm E Partronen T Härmä M Hublin C Lallukka T Peltonen M. Prevalence of insomnia-related symptoms continues to increase in the Finnish working-age population. J Sleep Res. (2016) 25:454–7. 10.1111/jsr.12398 26868677

[2] Li Y Qin Q Sun Q Sandford L D Vgnotzas AN Tang X. Insomnia and psychological reactions during the COVID-19 outbreak in China. J Clin Sleep Med. (2020) 16:1417–8. 10.5664/jcsm.8524 32351206 PMC7446072

[3] Kancherla BS Upender R Collen J F Rishi MA Sullivan SS Ahmed O. Sleep, fatigue and burnout among physicians: an American Academy of Sleep Medicine position statement. J Clin Sleep Med. (2020) 16:803–5. 10.5664/jcsm.8408 32108570 PMC7849815

[4] Robbins R Jackson CL Underwood P Vieira D Jean-Louis G Buxton OM. Employee sleep and workplace health promotion: a systematic review. Am J Health Promot. (2019) 33:1009–19. 10.1177/0890117119841407 30957509 PMC9035216

[5] McGregor A Ashbury F Caputi P Iverson D. A preliminary investigation of health and work-environment factors on presenteeism in the workplace. J Occup Environ Med. (2018) 60:e671–78. 10.1097/JOM.0000000000001480 30312220

[6] Seow LSE Tan XW Chong SA Vaingankar JA Abdin E Shafie S. Independent and combined associations of sleep duration and sleep quality with common physical and mental disorders: results from a multi-ethnic population-based study. PLoS ONE. (2020) 15-7. 10.1371/journal.pone.0235816 32673344 PMC7365445

[7] Åkerstedt T Mittendorfer-Rutz E Rahman S. Sleep disturbance and work-related mental strain: a national prospective cohort study of the prediction of subsequent long-term sickness absence, disability pension, and mortality. Scand J Public Health. (2020) 48:888–95. 10.1177/1403494820911813 32195635 PMC7678333

[8] Kessler RC Berglund PA Coulouvrat C Hajak G Roth T Shahly V. Insomnia and the performance of US workers: results from the America insomnia survey. Sleep. (2011) 34:1161–71. 10.5665/SLEEP.1230 21886353 PMC3157657

[9] Saksvik IB Bjorvatn B Hetland H Sandal GM Pallesen S. Individual differences in tolerance to shift work–a systematic review. Sleep Med Rev. (2011) 15:221–35. 10.1016/j.smrv.2010.07.002 20851006

[10] Theorell T Perski A Åkerstedt T Sigala F Ahlberg-Hultén G Svensson J. Changes in job strain in relation to changes in physiological state. A longitudinal study. Scand J Work Environ Health. (1988) 14:189–96. 10.5271/sjweh.1932 3393855

[11] Karasek R Brisson C Kawakami N Houtman I Bongers P Amick B. The Job Content Questionnaire (JCQ): an instrument for internationally comparative assessments of psychosocial job characteristics. J Occup Health Psychol. (1998) 3:322–55. 10.1037/1076-8998.3.4.322 9805280

[12] Swedish Council on Health Technology Assessment (SBU) Assessment. SBU Systematic Review Summaries. (2013). Available online at: https://www.sbu.se/work_sleepStockholm (accessed September 7, 2021).

[13] Kivimäki M Nyberg ST Batty GD Fransson EI Heikkilä K Alfredsson L. Job strain as a risk factor for coronary heart disease: a collaborative meta-analysis of individual participant data. Lancet. (2012) 380:1491–7. 10.1016/S0140-6736(12)60994-5 22981903 PMC3486012

[14] Lischewski D Zimmermann S Heimlich J Glos M Westermayer G Penzel T Fietze I. Industrial health Shift work and sleep disorders. Somnologie. (2011) 15:5–13. 10.1007/s11818-011-0502-4

[15] Litwiller B Snyder LA Taylor WD Steele LM. The relationship between sleep and work: a meta-analysis. J Appl Psychol. (2017) 102:682–99. 10.1037/apl0000169 27893255

[16] Westermayer G Stein B. Produktivitätsfaktor Betriebliche Gesundheit. Göttingen: Hogrefe-Verlag (2006).

[17] Strine TW Chapman DP. Associations of frequent sleep insufficiency with health-related quality of life and health behaviors. J Sleep Med. (2005) 6:23–7. 10.1016/j.sleep.2004.06.003 15680291

[18] Ducki A. Diagnose gesundheitsförderlicher Arbeit: eine Gesamtstrategie zur betrieblichen Gesundheitsanalyse. (2000). Zürich: Vdf Hochschulverlag an der ETH.

[19] Ducki A. Arbeits- und organisationspsychologische Gesundheitsanalysen—Entwicklung und Erprobung eines Befragungsinstrumentes im Rahmen eines Mehrebenen-Ansatzes zur betrieblichen Gesundheitsanalyse [Dissertation]. (1998). Leipzig: Universität Leipzig.

[20] Brand D. Gesundheitsförderliche und -gefährdende Arbeitsbedingungen und deren Einfluss auf die Gesundheit älterer Arbeitnehmer^*^innen ab 50 Jahren [Masterarbeit]. (2021). Berlin: Charité Universitätmedizin Berlin, Berlin School of Public health.

[21] Antonovsky A. Salutogenese: Zur Entmystifizierung der Gesundheit. In: Forum für Verhaltenstherapie und psychosoziale Praxis. (1997). Tübingen, Germany: dgvt-Verlag.

[22] La YK Choi YH Chu MK Nam JM ChoiY C Kim WJ. Gender differences influence over insomnia in Korean population: a cross-sectional study. PLoS ONE. (2020) 15:e0227190. 10.1371/journal.pone.0227190 31917784 PMC6952093

[23] Ohayon MM. Epidemiology of insomnia: what we know and what we still need to learn. Sleep Med Rev. (1997) 6:97–111. 10.1053/smrv.2002.0186 12531146

[24] Zhang B Wing YK. Sex differences in insomnia: a meta-analysis. Sleep. (2006) 29:85–93. 10.1093/sleep/29.1.85 16453985

[25] Gurubhagavatula I Barger LK Barnes CM Basner M Boivin DB Dawson D. Guiding principles for determining work shift duration and addressing the effects of work shift duration on performance, safety, and health: guidance from the American Academy of Sleep Medicine and the Sleep Research Society. Sleep. (2021) 44:zsab161. 10.1093/sleep/zsab161 34373924

[26] Magnavita N Garbarino S. Sleep, health and wellness at work: a scoping review. Int J Environ Res Public Health. (2017) 14:1347. 10.3390/ijerph14111347 29113118 PMC5707986

[27] Schierholz RS Darius S Böckelmann I. Associations of work-related strain with subjective sleep quality and individual daytime sleepiness. Dtsch Med Wochenschr. (2019) 144:e121–9. 10.1055/a-0873-7196 31067574

[28] Leitaru N Kremers S Hagberg J Björklund C Kwak L. Associations between job-strain, physical activity, health status, and sleep quality among Swedish municipality workers. J Occup Environ Med. (2019) 61:e56–60. 10.1097/JOM.0000000000001516 30540651 PMC6416142

[29] Takaki J Taniguchi T Fukuoka E Fujii Y Tsutsumi A Nakajima K. Workplace bullying could play important roles in the relationships between job strain and symptoms of depression and sleep disturbance. J Occup Health. (2010) 52:367–74. 10.1539/joh.L10081 20944438

[30] Halolnen JI Lallukka T Pentti J Stenholm S Rod NH Virtanen M. Change in job strain as a predictor of change in insomnia symptoms: analyzing observational data as a non-randomized pseudo-trial. Sleep. (2017) 40:zsw007. 10.1093/sleep/zsw007 28364463 PMC5806551

[31] Metlaine A Sauvet F Gomez-Merino D Elbaz M Delafosse JY Leger D. Association between insomnia symptoms, job strain and burnout syndrome: a cross-sectional survey of 1300 financial workers. BMJ Open. (2017) 7:e012816. 10.1136/bmjopen-2016-012816 28087546 PMC5253603

[32] Åkerstedt T Narusyte J Svedberg P. Night work, mortality, and the link to occupational group and sex. Scand J Work Environ Health. (2017) 46:508. 10.5271/sjweh.3892 32270204 PMC7737802

[33] Brossoit RM Crain TL Hammer LB Lee S Bodner TE Buxton OM. Associations among patient care workers' schedule control, sleep, job satisfaction and turnover intentions. Stress Health. (2021) 36:442–56. 10.1002/smi.2941 32181575 PMC8919502

[34] Kecklund G Eriksen CA Åkerstedt T. Police officers attitude to different shift systems: association with age, present shift schedule, health and sleep/wake complaints. Appl Ergon. (2008) 39:565–71. 10.1016/j.apergo.2008.01.002 18281011

[35] Karhula K Hakola T Koskinen A Lallukka T Ojajärvi A Puttonen S. Ageing shift workers' sleep and working-hour characteristics after implementing ergonomic shift-scheduling rules. J Sleep Res. (2021) 30:e13227. 10.1111/jsr.13227 33166038 PMC8365717

[36] Howarth A Quesada J Silva J Judycki S Mills PR. The impact of digital health interventions on health-related outcomes in the workplace: a systematic review. Digit Health. (2021) 4:2055207618770861. 10.1177/2055207618770861 29942631 PMC6016571

[37] Lerman SE Eskin E Flower DJ George EC Gerson B Hartenbaum N Hursh SR Moore-Ede M. Fatigue risk management in the workplace. J Occup Environ Med. 54:231–58. 10.1097/JOM.0b013e318247a3b0 22269988

[38] Magnavita N. La gestione del rischio dei disturbi del sonno mediante il metodo A.S.I.A. G. Ital. Med. Lav. Ergon. (2012) 35:2.

[39] Neil-Sztramko SE Pahwa M Demers PA Gotay CC. Health-related interventions among night shift workers: a critical review of the literature. Scand J Work Environ Health. (2014) 40:543–56. 10.5271/sjweh.3445 24980289

[40] Dingel JI Neiman B. How many jobs can be done at home? J Public Econ. (2020) 189:104235. 10.1016/j.jpubeco.2020.104235 32834177 PMC7346841

[41] Olson R Crain TL Bodner TE King R Hammer LB Klein LC. A workplace intervention improves sleep: results from the randomized controlled Work, Family, and Health Study. Sleep Health. (2015) 1:55–65. 10.1016/j.sleh.2014.11.003 29073416 PMC9019820

