# Supplementary material for: The Interplay Between Poor Sleep and Work-Related Health
Source: Front Public Health. 2022 Jul 7;10:866750. doi: 10.3389/fpubh.2022.866750 (PMC9301247; doi:10.3389/fpubh.2022.866750)
Supplement: Supplementary file 1 [file Table_1.DOCX]

Supplementary Material

|  |  |  |  |  |  |  |  |
| --- | --- | --- | --- | --- | --- | --- | --- |
| *Type: health potential (HP), health risk (HR), health indicator (HI)* | | | | | | **criteria of reliability (internal consistence)** | |
| **Type** | **Scale name** | **No. of items** | **Item examples** | **Description** | **Origin** | ***Cronbach's α*** | ***coefficients of discrimination*** |
| HI | Joy of work | 5 | My work is fun. There are days when I feel proud about what I have done at work. | Measures the extent to which work is an object of joy, pride and purpose. | Ducki (1998, 2000) | 0.89 | 0.66 - 0.79 |
|  | Confidence | 3 | If unexpected problems arise at work, I don’t cope well with them. I always know how I should behave if an unexpected situation arises at work. | Measures to what extent one has a feeling of control over dealing with difficult or unexpected events through one's own actions. | Ducki (1998, 2000), based on Schwarzer (1994); modified by BGF GmbH, based on Schyns & von Collani (1999) | 0.62 | 0.35 - 0.51 |
|  | Psychological impairments | 11 | I have problems sleeping (falling asleep, staying asleep). Sometimes I think it is just not worth getting upset anymore. | Measures the extent to which psychological impairments like symptoms of exhaustion or irritability are perceived. | Mohr (1991), modified by Ducki (1998, 2000) | 0.90 | 0.48 - 0.76 |
|  | Physical impairments | 16 | I have pain in my joints. I have headaches. | Measures the extent to which physical impairments are perceived. |  | 0.87 | 0.36 - 0.61 |
|  | Information & participation in the company | 5 | Staff are sufficiently informed about upcoming changes and decisions. In our company, personal initiative and commitment are welcomed. | Measures the extent to which information and participation is given at the overall company level. | Ducki (1998, 2000), modified and expanded by BGF GmbH | 0.81 | 0.53 - 0.69 |
| HP | Information & participation at the workplace | 7 | I receive up-to-date and sufficient responses from my superior about the outcomes of my work. My personal suggestions receive reasonable consideration. | Measures the extent to which information and participation is given with regard to the specific workplace or work tasks. | developed by BGF GmbH | 0.90 | 0.66 - 0.79 |
|  | Scope of decision making | 8 | I have various possibilities to complete the tasks I am working on. I can always decide anew, which course of action is the best to take. | Measures to what extent the work tasks can be planned, decided and carried out independently. | Ducki (1998, 2000), modified and expanded by BGF GmbH | 0.94 | 0.69 - 0.84 |
|  | Fair assessment | 4 | My superior appraises my performance in a fair manner. Some colleagues are favored by my superior. | Measures the extent to which superiors treat and assess employees fairly. | developed by BGF GmbH | 0.81 | 0.62 - 0.67 |
|  | Appreciation | 4 | I am satisfied with my pay. My superior acknowledges good performance. | Measures the extent to which work performed is appropriately assessed, remunerated, and appreciated. | Ducki (1998, 2000), modified and expanded by BGF GmbH | 0.73 | 0.41 - 0.63 |
|  | Learning at work | 3 | In this work, I can always learn something new. I can develop my skills and abilities in my work. | Measures to what extent abilities and skills can be applied, developed, and new things can be learned. | Ducki (1998, 2000), modified and expanded by BGF GmbH | 0.75 | 0.49 - 0.67 |
|  | Work organization | 8 | My areas of responsibility and competence are clearly defined. Work processes in my work area are well organized. | Measures the extent to which competencies, responsibilities and the general process organization are regulated. | Ducki (1998, 2000), modified and expanded by BGF GmbH | 0.86 | 0.40 - 0.68 |
| HP | Development opportunities | 3 | Our company offers good vocational and educational training opportunities. Our company provides staff with good opportunities for promotion. | Measures to what extent the company offers opportunities for career advancement and development. | Ducki (1998, 2000), modified and expanded by BGF GmbH | 0.83 | 0.59 - 0.78 |
|  | Identification | 4 | Our products and services are attractive for our clients/customers. Our company has a good public reputation. | Measures the extent to which employees identify with the company and the products/services. | Ducki (1998, 2000) | 0.84 | 0.68 - 0.71 |
|  | Leadership | 10 | My superior is open to constructive criticism. My superior radiates calmness and maintains his/her composure, even in difficult times. | Measures the extent to which it is possible to communicate with superiors in an atmosphere of openness, trust and support- | developed by BGF GmbH (Bonn, 2002) | 0.95 | 0.72 - 0.85 |
|  | Working climate | 3 | I can openly talk about all important matters with my immediate colleagues. Anyone who addresses problems or difficulties makes him/herself immediately unpopular. | Measures the extent to which there is a trusting interpersonal relationship between colleagues. | Ducki (1998, 2000), modified by BGF GmbH | 0.74 | 0.46 - 0.63 |
|  | | | | | | | |
|  | Technical support from leadership | 4 | My superior can explain specialized work-related questions in a comprehensible manner. My superior is always open to my specialized work-related questions. | Measures the extent to which superiors are available to support employees with technical questions and problems. | developed by BGF GmbH (Bonn, 2002) | 0.93 | 0.81 - 0.85 |
| HP |  |  |  |  |  |  |  |
| HR | Time pressure | 3 | Generally, there is sufficient time available for me, to carefully plan my work tasks. It happens that I stay longer to do my work. | Measures the extent to which there is enough time to plan and complete the work tasks. | developed by BGF GmbH | 0.77 | 0.50 - 0.70 |
|  | Interruptions | 4 | It is the case that I am interrupted in my work, by defective or unsuitable equipment, tools or machines. It happens that my work is interrupted, because information or documents are missing. | Measures to what extent work has to be interrupted due to faulty/missing materials, information, work equipment or people. | Ducki (1998, 2000), modified by BGF GmbH | 0.75 | 0.35 - 0.67 |
|  | Technical overload | 2 | It occurs that work tasks are given to me, for which I am not sufficiently prepared. It happens that work is passed on to me, which exceeds my level of competence. | Measures to what extent the employees feel they are up to the tasks assigned. | developed by BGF GmbH (Beck, 2003, based on Rimann & Udris, 1993) | 0.70 | 0.55 |
|  | Job insecurity | 4 | I will probably need to take a worse position in my company. My job is secure. | Measures the extent to which job insecurity exists - both in general and with regard to internal restructuring. | Ducki (1998, 2000), based on Semmer & Dunckel (1991) | 0.77 | 0.55 - 0.65 |
|  | Physical-environmental stress | 12 | In my workspace the following factors often disturb/impair me: - Cold - Noise | Measures the extent to which employees perceive non-task-specific physical-environmental conditions. | Ducki (1998, 2000), many similar compilations exist | 0.88 | 0.44 - 0.67 |
|  | Ergonomic-environmental stress | 4 | In my workspace the following factors often disturb/impair me: - unsuitable tables/worktops - unsuitable computers/monitors | Measures the extent to which employees perceive non-task-specific ergonomic-environmental conditions. | Ducki (1998, 2000), many similar compilations exist | 0.79 | 0.52 - 0.66 |
|  | Physical strain | 8 | In my workspace the following factors often disturb/impair me: - standing for long periods - lifting/carrying | Measures the extent to which physical strains occur when performing the work tasks. | developed by BGF GmbH, many similar compilations exist | 0.88 | 0.25- 0.81 |

Supplementary Table 1: A table to show each scale used in the survey. Each scale contains information regarding information, origin, Cronbach's α, and coefficients of discrimination.

| ***I often have sleep disorders (trouble falling asleep and/or staying asleep).*** | ***Completely agree*** | | ***Mostly agree*** | | ***Partly agree*** | | ***Mostly disagree*** | | ***Completely disagree*** | | ***SUM*** |
| --- | --- | --- | --- | --- | --- | --- | --- | --- | --- | --- | --- |
|  | **N** | ***%*** | **N** | ***%*** | **N** | ***%*** | **N** | ***%*** | **N** | ***%*** | **N** |
| ***Male*** | **576** | ***8.9*** | **799** | ***12.4*** | **1511** | ***23.5*** | **1632** | ***25.3*** | **1920** | ***29.8*** | **6438** |
| ***Up to 29 years*** | 103 | *9.9* | 137 | *13.2* | 203 | *19.5* | 253 | *24.4* | 343 | *33.0* | 1039 |
| ***30 - 39 years*** | 130 | *9.1* | 173 | *12.1* | 337 | *23.5* | 336 | *23.4* | 457 | *31.9* | 1433 |
| ***40 - 49 years*** | 168 | *9.6* | 197 | *11.3* | 443 | *25.4* | 469 | *26.9* | 467 | *26.8* | 1744 |
| ***50 years and older*** | 156 | *8.2* | 252 | *13.3* | 461 | *24.4* | 481 | *25.4* | 543 | *28.7* | 1893 |
| ***N.a.*** | 19 | *5.8* | 40 | *12.2* | 67 | *20.4* | 93 | *28.3* | 110 | *33.4* | 329 |
| ***Female*** | **1417** | ***13.7*** | **1424** | ***13.8*** | **2681** | ***26.0*** | **2186** | ***21.2*** | **2601** | ***25.2*** | **10309** |
| ***Up to 29 years*** | 145 | *10.8* | 205 | *15.3* | 312 | *23.2* | 329 | *24.5* | 351 | *26.2* | 1342 |
| ***30 - 39 years*** | 206 | *10.3* | 231 | *11.6* | 483 | *24.2* | 468 | *23.4* | 609 | *30.5* | 1997 |
| ***40 - 49 years*** | 421 | *13.6* | 405 | *13.0* | 831 | *26.8* | 616 | *19.8* | 832 | *26.8* | 3105 |
| ***50 years and older*** | 574 | *17.6* | 507 | *15.6* | 907 | *27.8* | 624 | *19.2* | 646 | *19.8* | 3258 |
| ***N.a.*** | 71 | *11.7* | 76 | *12.5* | 148 | *24.4* | 149 | *24.5* | 163 | *26.9* | 607 |
| ***N.a.*** | 309 | *11.2* | 340 | *12.3* | 652 | *23.6* | 625 | *22.7* | 831 | *30.1* | 2757 |
| *Up to 29 years* | 20 | *8.6* | 32 | *13.7* | 39 | *16.7* | 63 | *27.0* | 79 | *33.9* | 233 |
| *30 - 39 years* | 48 | *11.9* | 45 | *11.2* | 93 | *23.1* | 87 | *21.6* | 129 | *32.1* | 402 |
| *40 - 49 years* | 60 | *9.7* | 68 | *11.0* | 149 | *24.2* | 137 | *22.2* | 202 | *32.8* | 616 |
| *50 years and older* | 62 | *11.9* | 73 | *14.0* | 133 | *25.5* | 113 | *21.6* | 141 | *27.0* | 522 |
| *N.a.* | 119 | *12.1* | 122 | *12.4* | 238 | *24.2* | 225 | *22.9* | 280 | *28.5* | 984 |
| ***Sample in total*** | **2302** | **11.8** | **2563** | **13.1** | **4844** | **24.8** | **4443** | **22.8** | **5352** | **27.4** | **19504** |
| ***Up to 29 years*** | 268 | *10.3* | 374 | *14.3* | 554 | *21.2* | 645 | *24.7* | 773 | *29.6* | 2614 |
| ***30 - 39 years*** | 384 | *10.0* | 449 | *11.7* | 913 | *23.8* | 891 | *23.3* | 1195 | *31.2* | 3832 |
| ***40 - 49 years*** | 649 | *11.9* | 670 | *12.3* | 1,423 | *26.0* | 1222 | *22.4* | 1501 | *27.5* | 5465 |
| ***50 years and older*** | 792 | *14.0* | 832 | *14.7* | 1,501 | *26.5* | 1218 | *21.5* | 1330 | *23.4* | 5673 |
| ***N.a.*** | 209 | *10.9* | 238 | *12.4* | 453 | *23.6* | 467 | *24.3* | 553 | *28.8* | 1920 |

Supplementary Table 2: A table to show the frequency of respondents for the scale regarding the question on sleep. These responses were then split by age and gender.

|  | **Moderate/severe sleep problems** | | **Little/no sleep problems** | |
| --- | --- | --- | --- | --- |
| **Industry** | **N** | **%** | **N** | **%** |
| **Male total** | **1375** | **21.4** | **3552** | **55.2** |
| Manufacturing | 317 | 23.2 | 691 | 50.7 |
| Financial and insurance services | 195 | 21.5 | 496 | 54.6 |
| Economic business services (e.g. call center, facility management, personnel services) | 229 | 23. | 532 | 53.2 |
| Public administration, social insurance, defense | 120 | 19.1 | 364 | 57.8 |
| Education | 27 | 12.9 | 139 | 66.5 |
| Health and social services | 210 | 22. | 530 | 55.6 |
| Other Services (e.g. associations, advocacies; repairs; personal services) | 125 | 19.4 | 388 | 60.1 |
| Other branches of industry | 152 | 20.7 | 412 | 56.21 |
| **Female total** | **2841** | **27.6** | **4787** | **46.4** |
| Manufacturing | 243 | 35.5 | 264 | 38.6 |
| Financial and insurance services | 476 | 30.4 | 658 | 42 |
| Economic business services (e.g. call center, facility management, personnel services) | 410 | 28.1 | 680 | 46.5 |
| Public administration, social insurance, defense | 372 | 22.9 | 810 | 49.9 |
| Education | 67 | 16.8 | 239 | 59.8 |
| Health and social services | 911 | 30.5 | 1313 | 43.9 |
| Other Services (e.g. associations, advocacies; repairs; personal services) | 241 | 22.3 | 580 | 53.7 |
| Other branches of industry | 121 | 24.3 | 243 | 48.8 |
|  | **Moderate/severe sleep problems** | | **Little/no sleep problems** | |
| **Industry** | **N** | **%** | **N** | **%** |
|  |  |  |  |  |
| **NA total** | **649** | **23.5** | **1456** | **52.1** |
| Manufacturing | 62 | 22.6 | 140 | 51.1 |
| Financial and insurance services | 3 | 42.9 | 2 | 28.6 |
| Economic business services (e.g. call center, facility management, personnel services) | 65 | 35.7 | 83 | 45.6 |
| Public administration, social insurance, defense | 203 | 21.4 | 514 | 54.2 |
| Education | 65 | 19.1 | 186 | 54.7 |
| Health and social services | 123 | 27.7 | 220 | 49.6 |
| Other Services (e.g. associations, advocacies; repairs; personal services) | 22 | 30.1 | 33 | 45.2 |
| Other branches of industry | 106 | 21.7 | 278 | 56.9 |
| **Sample in total** | **4865** | **24.9** | **9795** | **50.2** |
| Manufacturing | 622 | 26.8 | 1095 | 47.2 |
| Financial and insurance services | 674 | 27.2 | 1156 | 46.6 |
| Economic business services (e.g. call center, facility management, personnel services) | 704 | 26.7 | 1295 | 49.1 |
| Public administration, social insurance, defense | 695 | 21.7 | 1688 | 52.7 |
| Education | 159 | 16.8 | 564 | 59.4 |
| Health and social services | 1244 | 28.3 | 2063 | 47 |
| Other Services (e.g. associations, advocacies; repairs; personal services) | 388 | 21.6 | 1001 | 55.6 |
| Other branches of industry | 379 | 22 | 933 | 54.2 |

Supplementary Table 3: A table to show the total and gender specific frequency of good and poor sleepers in each industry.

|  | Health Aspect | Variation | **Sum of Squares** | **df** | **Mean of Squares** | **F** | **Sig.** |
| --- | --- | --- | --- | --- | --- | --- | --- |
| Health indicators | Joy of work | Between groups | 306138.524 | 1 | 306138.524 | 638.863 | 0.00 |
|  |  | Within groups | 6995258.934 | 14598 | 479.193 |  |  |
|  |  | Total | 7301397.458 | 14599 |  |  |  |
|  | Confidence | Between groups | 60342.069 | 1 | 60342.069 | 223.782 | 0.00 |
|  |  | Within groups | 3917422.400 | 14528 | 269.646 |  |  |
|  |  | Total | 3977764.469 | 14529 |  |  |  |
|  | Psychological impairments | Between groups | 3316016.547 | 1 | 3316016.547 | 12689.774 | 0.00 |
|  |  | Within groups | 3830341.746 | 14658 | 261.314 |  |  |
|  |  | Total | 7146358.293 | 14659 |  |  |  |
|  | Physical impairments | Between groups | 1413076.294 | 1 | 1413076.294 | 5555.150 | 0.00 |
|  |  | Within groups | 3725028.134 | 14644 | 254.372 |  |  |
|  |  | Total | 5138104.427 | 14645 |  |  |  |
| Health potentials | Identification | Between groups | 170595.208 | 1 | 170595.208 | 379.837 | 0.00 |
|  |  | Within groups | 6499316.684 | 14471 | 449.127 |  |  |
|  |  | Total | 6669911.893 | 14472 |  |  |  |
|  | Information & participation in the company | Between groups | 271164.042 | 1 | 271164.042 | 565.251 | 0.00 |
|  |  | Within groups | 6979493.705 | 14549 | 479.723 |  |  |
|  |  | Total | 7250657.748 | 14550 |  |  |  |
|  | Information & participation at the workplace | Between groups | 321318.289 | 1 | 321318.289 | 597.693 | 0.00 |
|  |  | Within groups | 7853219.615 | 14608 | 537.597 |  |  |
|  |  | Total | 8174537.904 | 14609 |  |  |  |
|  | Work organization | Between groups | 231625.693 | 1 | 231625.693 | 613.316 | 0.00 |
|  |  | Within groups | 5517627.175 | 14610 | 377.661 |  |  |
|  |  | Total | 5749252.868 | 14611 |  |  |  |
|  | Scope of decision making | Between groups | 355184.700 | 1 | 355184.700 | 496.543 | 0.00 |
|  |  | Within groups | 10356325.035 | 14478 | 715.315 |  |  |
|  |  | Total | 10711509.735 | 14479 |  |  |  |
|  | Learning at work | Between groups | 427142.704 | 1 | 427142.704 | 700.110 | 0.00 |
|  |  | Within groups | 8877684.687 | 14551 | 610.108 |  |  |
|  |  | Total | 9304827.390 | 14552 |  |  |  |
|  | Development opportunities | Between groups | 181305.320 | 1 | 181305.320 | 263.305 | 0.00 |
|  |  | Within groups | 9870718.255 | 14335 | 688.575 |  |  |
|  |  | Total | 10052023.575 | 14336 |  |  |  |
|  | Appreciation | Between groups | 361527.916 | 1 | 361527.916 | 647.433 | 0.00 |
|  |  | Within groups | 8145967.857 | 14588 | 558.402 |  |  |
|  |  | Total | 8507495.772 | 14589 |  |  |  |
|  |  | | | | | | |
|  | Health Aspect | Variation | **Sum of Squares** | **df** | **Mean of Squares** | **F** | **Sig.** |
|  | Fair assessment | Between groups | 540317.575 | 1 | 540317.575 | 950.858 | 0.00 |
|  |  | Within groups | 8229284.883 | 14482 | 568.242 |  |  |
|  |  | Total | 8769602.458 | 14483 |  |  |  |
|  | Leadership | Between groups | 343169.986 | 1 | 343169.986 | 551.799 | 0.00 |
|  |  | Within groups | 9042596.927 | 14540 | 621.912 |  |  |
|  |  | Total | 9385766.913 | 14541 |  |  |  |
|  | Technical support from leadership | Between groups | 248697.063 | 1 | 248697.063 | 383.302 | 0.00 |
|  |  | Within groups | 9117340.579 | 14052 | 648.829 |  |  |
|  |  | Total | 9366037.642 | 14053 |  |  |  |
|  | Technical exchange with colleagues | Between groups | 286339.576 | 1 | 286339.576 | 447.931 | 0.00 |
|  |  | Within groups | 7881310.575 | 12329 | 639.250 |  |  |
|  |  | Total | 8167650.151 | 12330 |  |  |  |
|  | Working climate | Between groups | 475984.940 | 1 | 475984.940 | 806.944 | 0.00 |
|  |  | Within groups | 8588379.858 | 14560 | 589.861 |  |  |
|  |  | Total | 9064364.798 | 14561 |  |  |  |
| Health hazards | Time pressure | Between groups | 400099.392 | 1 | 400099.392 | 641.892 | 0.00 |
|  |  | Within groups | 9050502.221 | 14520 | 623.313 |  |  |
|  |  | Total | 9450601.613 | 14521 |  |  |  |
|  | Interruptions | Between groups | 309034.654 | 1 | 309034.654 | 667.297 | 0.00 |
|  |  | Within groups | 6728119.109 | 14528 | 463.114 |  |  |
|  |  | Total | 7037153.763 | 14529 |  |  |  |
|  | Technical overload | Between groups | 212611.753 | 1 | 212611.753 | 519.397 | 0.00 |
|  |  | Within groups | 5915013.181 | 14450 | 409.343 |  |  |
|  |  | Total | 6127624.934 | 14451 |  |  |  |
|  | Job insecurity | Between groups | 227704.794 | 1 | 227704.794 | 360.365 | 0.00 |
|  |  | Within groups | 8212456.023 | 12997 | 631.873 |  |  |
|  |  | Total | 8440160.817 | 12998 |  |  |  |
|  | Physical-environmental stress | Between groups | 597183.797 | 1 | 597183.797 | 1181.843 | 0.00 |
|  |  | Within groups | 7345023.970 | 14536 | 505.299 |  |  |
|  |  | Total | 7942207.767 | 14537 |  |  |  |
|  | Ergonomic-environmental stress | Between groups | 410553.404 | 1 | 410553.404 | 649.623 | 0.00 |
|  |  | Within groups | 8657588.709 | 13699 | 631.987 |  |  |
|  |  | Total | 9068142.113 | 13700 |  |  |  |
|  | Physical strain | Between groups | 571772.787 | 1 | 571772.787 | 942.822 | 0.00 |
|  |  | Within groups | 7757083.890 | 12791 | 606.449 |  |  |
|  |  | Total | 8328856.677 | 12792 |  |  |  |

Supplementary Table 4: A table to show the ANOVA for all scales in good and poor sleepers

|  | *HI = Health indicators HP = Health potentials HR = Health hazards* |  | | | | | |
| --- | --- | --- | --- | --- | --- | --- | --- |
|  |  | **Little/No Sleep Problems (N = 9,795)** | | | **Moderate/Severe Sleep Problems (N = 4,865)** | | |
|  |  | N | Mean | SD | N | Mean | SD |
| HI | Physical impairments | 9788 | 17.2 | 13.7 | 4858 | 38.0 | 19.7 |
|  | Psychological impairments | 9795 | 21.7 | 14.9 | 4865 | 53.6 | 18.4 |
|  | Confidence | 9716 | 76.4 | 15.7 | 4814 | 72.0 | 17.8 |
|  | Joy of work | 9750 | 71.2 | 20.9 | 4850 | 61.5 | 23.8 |
| HP | Working climate | 9733 | 63.4 | 23.6 | 4829 | 51.2 | 25.6 |
|  | Technical exchange with colleagues | 8262 | 66.3 | 24.7 | 4069 | 56.0 | 26.5 |
|  | Technical support from leadership | 9372 | 71.8 | 24.5 | 4682 | 62.9 | 27.3 |
|  | Leadership | 9725 | 65.8 | 24.2 | 4817 | 55.5 | 26.4 |
|  | Fair assessment | 9674 | 76.2 | 22.4 | 4810 | 63.2 | 26.5 |
|  | Appreciation | 9751 | 53.6 | 23.7 | 4839 | 43.0 | 23.4 |
|  | Development opportunities | 9555 | 46.1 | 26.6 | 4782 | 38.6 | 25.6 |
|  | Learning at work | 9728 | 64.6 | 24.3 | 4825 | 53.1 | 25.4 |
|  | Scope of decision making | 9695 | 60.7 | 26.3 | 4785 | 50.2 | 27.6 |
|  | work organization | 9767 | 65.5 | 18.9 | 4845 | 57.0 | 20.4 |
|  | Information & participation at the workplace | 9763 | 64.3 | 22.8 | 4847 | 54.3 | 24.0 |
|  | Information & participation in the company | 9724 | 46.9 | 22.3 | 4827 | 37.7 | 21.1 |
|  | Identification | 9670 | 71.9 | 20.3 | 4803 | 64.6 | 22.9 |
| HH | Physical strain | 8455 | 27.6 | 23.7 | 4338 | 41.7 | 26.4 |
|  | Ergonomic-environmental stress | 9182 | 21.9 | 23.5 | 4519 | 33.5 | 28.1 |
|  | Physical-environmental stress | 9724 | 28.1 | 21.5 | 4814 | 41.7 | 24.4 |
|  | Job insecurity | 8693 | 23.8 | 23.6 | 4306 | 32.7 | 27.9 |
|  | Technical overload | 9677 | 21.3 | 18.9 | 4775 | 29.5 | 22.6 |
|  | Interruptions | 9720 | 35.3 | 20.8 | 4810 | 45.1 | 22.9 |
|  | Time pressure | 9717 | 45.5 | 24.6 | 4805 | 56.6 | 25.7 |

Supplementary Table 5: A table to show the mean and standard deviation of each scale answered by good and poor sleepers. The total number of missing participants for each scale can be calculated by subtracting the total number of participants in each group (N = 4,865 poor sleepers, N = 9,795 good sleepers) with the total number of participants in each group for every scale in Supplementary Table 5.

| **Outcome: Health Index** | **Moderate/Severe Sleeping Problems** | | | | | | | |
| --- | --- | --- | --- | --- | --- | --- | --- | --- |
|  | **Model 1^a^** | | R²: 39.0% N = 2,975 | | **Model 2^b^** | | R²: 39.4% N = 2,456 | |
|  | β | 95%CI | | p | β | 95%CI | | p |
|  |  | low | high |  |  | low | high |  |
| **Technical overload** *(i.e. not adequately prepared for the tasks)* | -0,501 | -0,582 | -0,420 | <0,001 | -0,525 | -0,616 | -0,434 | <0,001 |
| **Identification** *(i.e. being convinced of the company's products/services; company has a future)* | 0,424 | 0,346 | 0,502 | <0,001 | 0,463 | 0,373 | 0,554 | <0,001 |
| **Learning at work** *(i.e. developing skills & abilities)* | 0,427 | 0,353 | 0,502 | <0,001 | 0,396 | 0,313 | 0,479 | <0,001 |
| **Physical-environmental stress** *(i.e. exposure to dust, drafts)* | -0,283 | -0,365 | -0,201 | <0,001 | -0,272 | -0,364 | -0,180 | <0,001 |
| **Scope of decision making** *(i.e. planning task independently; making a lot of own decisions)* | 0,196 | 0,128 | 0,264 | <0,001 | 0,237 | 0,161 | 0,314 | <0,001 |
| **Job insecurity** *(i.e. taking on a lesser job within the company)* | -0,143 | -0,206 | -0,081 | <0,001 | -0,181 | -0,250 | -0,112 | <0,001 |
| **Work organization** *(i.e. work processes are well organized)* | 0,133 | 0,017 | 0,248 | 0,024 | 0,138 | 0,011 | 0,264 | 0,033 |
| **Technical exchange with colleagues** *(i.e. exchange between colleagues is possible)* | 0,113 | 0,043 | 0,184 | 0,002 | 0,139 | 0,060 | 0,218 | 0,001 |
| **Time pressure** *(i.e. not enough time to carefully plan the tasks)* | -0,134 | -0,204 | -0,063 | <0,001 | -0,120 | -0,199 | -0,040 | 0,003 |
| **Ergonomic-environmental stress** *(i.e. unsuitable seating/office chairs)* | -0,116 | -0,179 | -0,052 | <0,001 | -0,089 | -0,162 | -0,016 | 0,016 |
| **Working climate** *(i.e. addressing difficulties or problems without becoming unpopular)* | 0,100 | 0,026 | 0,173 | 0,008 | 0,099 | 0,017 | 0,182 | 0,018 |
| **Development opportunities**  *(i.e. good training opportunities)* | 0,102 | 0,028 | 0,176 | 0,007 | 0,098 | 0,016 | 0,181 | 0,019 |

β: non standardized regression coefficient; 95%CI: confidence interval; p: p-value; R²: adjusted explained variation; N = number of cases/persons included in the analysis. For the sake of clarity, only significant results (p < 0.05) are shown, but the R² relates to the complete models with all included independent variables. (black font = significant influence). ^a^ multiple linear regression (mlr) with the Health Index as the outcome (dependent variable) and all work related potentials and risks as exposition (20 independent variables). The criteria for the mlr were all met. ^b^ like ^a^ but additionally adjusted for age, sex, and branch of industry.

Supplementary Table 6: A table to show the multiple linear regression with the health index as the outcome for poor sleepers

| **Outcome: Health Index** | **Little/No Sleeping Problems** | | | | | | | |
| --- | --- | --- | --- | --- | --- | --- | --- | --- |
|  | **Model 1^a^** | | R²: 43.1% N = 5,898 | | **Model 2^b^** | | R²: 44.0% N = 4,659 | |
|  | β | 95%CI | | p | β | 95%CI | | p |
|  |  | low | high |  |  | low | high |  |
| **Technical overload** *(i.e. not adequately prepared for the tasks)* | -0,523 | -0,578 | -0,467 | <0,001 | -0,541 | -0,603 | -0,479 | <0,001 |
| **Identification** *(i.e. being convinced of the company's products/services; company has a future)* | 0,456 | 0,403 | 0,510 | <0,001 | 0,480 | 0,419 | 0,542 | <0,001 |
| **Learning at work** *(i.e. developing skills & abilities)* | 0,485 | 0,438 | 0,532 | <0,001 | 0,483 | 0,429 | 0,536 | <0,001 |
| **Physical-environmental stress** *(i.e. exposure to dust, drafts)* | -0,173 | -0,228 | -0,117 | <0,001 | -0,178 | -0,240 | -0,117 | <0,001 |
| **Scope of decision making** *(i.e. planning task independently; making a lot of own decisions)* | 0,121 | 0,078 | 0,163 | <0,001 | 0,110 | 0,061 | 0,159 | <0,001 |
| **Job insecurity** *(i.e. taking on a lesser job within the company)* | -0,111 | -0,154 | -0,067 | <0,001 | -0,122 | -0,170 | -0,074 | <0,001 |
| **Work organization** *(i.e. work processes are well organized)* | 0,118 | 0,043 | 0,192 | 0,002 | 0,111 | 0,030 | 0,192 | 0,007 |
| **Technical exchange with colleagues** *(i.e. exchange between colleagues is possible)* | 0,100 | 0,057 | 0,144 | <0,001 | 0,098 | 0,048 | 0,149 | <0,001 |
| **Time pressure** *(i.e. not enough time to carefully plan the tasks)* | -0,143 | -0,186 | -0,100 | <0,001 | -0,119 | -0,167 | -0,070 | <0,001 |
| **Ergonomic-environmental stress** *(i.e. unsuitable seating/office chairs)* | -0,051 | -0,096 | -0,006 | 0,025 | -0,052 | -0,104 | -0,001 | 0,047 |
| **Physical strain** *(i.e. working in a constrained posture)* | -0,126 | -0,172 | -0,080 | <0,001 | -0,089 | -0,142 | -0,036 | 0,001 |

β: non standardized regression coefficient; 95%CI: confidence interval; p: p-value; R²: adjusted explained variation; N = number of cases/persons included in the analysis. For the sake of clarity, only significant results (p < 0.05) are shown, but the R² relates to the complete models with all included independent variables. (black font = significant influence). ^a^ multiple linear regression (mlr) with the Health Index as the outcome (dependent variable) and all work related potentials and risks as exposition (20 independent variables). The criteria for the mlr were all met. ^b^ like ^a^ but additionally adjusted for age, sex, and branch of industry.

Supplementary Table 7: A table to show the multiple linear regression with the health index as the outcome for good sleepers

| **Outcome: Physical impairments** | **Moderate/Severe Sleeping Problems** | | | | | | | |
| --- | --- | --- | --- | --- | --- | --- | --- | --- |
|  | **Model 1^a^** | | R²: 27.3% N = 2,984 | | **Model 2^b^** | | R²: 30.9% N = 2,460 | |
|  | β | 95%CI | | p | β | 95%CI | | p |
|  |  | low | high |  |  | low | high |  |
| **Identification** *(i.e. being convinced of the company's products/services; company has a future)* | -0,033 | -0,062 | -0,003 | 0,028 | -0,036 | -0,070 | -0,003 | 0,034 |
| **Learning at work** *(i.e. developing skills & abilities)* | -0,046 | -0,074 | -0,018 | 0,001 | -0,048 | -0,079 | -0,018 | 0,002 |
| **Ergonomic-environmental stress** *(i.e. unsuitable seating/office chairs)* | 0,056 | 0,032 | 0,080 | <0,001 | 0,048 | 0,021 | 0,075 | <0,001 |
| **Physical-environmental stress** *(i.e. exposure to dust, drafts)* | 0,182 | 0,151 | 0,213 | <0,001 | 0,188 | 0,154 | 0,222 | <0,001 |
| **Technical overload** *(i.e. not adequately prepared for the tasks)* | 0,103 | 0,072 | 0,134 | <0,001 | 0,121 | 0,087 | 0,155 | <0,001 |
| **Physical strain** *(i.e. working in a constrained posture)* | 0,101 | 0,075 | 0,127 | <0,001 | 0,098 | 0,070 | 0,127 | <0,001 |
| **Fair assessment** *(i.e. superior does not favor certain colleagues)* | -0,049 | -0,084 | -0,013 | 0,007 | -0,069 | -0,108 | -0,030 | 0,001 |
| **Job insecurity** *(i.e. taking on a lesser job within the company)* | 0,058 | 0,035 | 0,082 | <0,001 | 0,067 | 0,041 | 0,092 | <0,001 |
| **Technical exchange with colleagues** *(i.e. exchange between colleagues is possible)* | -0,038 | -0,064 | -0,011 | 0,005 | -0,042 | -0,071 | -0,013 | 0,005 |
| **Time pressure** *(i.e. not enough time to carefully plan the tasks)* | 0,039 | 0,013 | 0,066 | 0,004 | 0,024 | -0,005 | 0,053 | 0,110 |
| **Leadership** *(i.e. superior makes clear and comprehensible decisions in difficult situations)* | 0,070 | 0,026 | 0,115 | 0,002 | 0,080 | 0,032 | 0,127 | 0,001 |
| **Working climate** *(i.e. addressing difficulties or problems without becoming unpopular)* | -0,052 | -0,080 | -0,024 | <0,001 | -0,040 | -0,071 | -0,010 | 0,009 |
| **Development opportunities** | -0,015 | -0,043 | 0,013 | 0,293 | -0,031 | -0,062 | -0,001 | 0,042 |

β: non standardized regression coefficient; 95%CI: confidence interval; p: p-value; R²: adjusted explained variation; N = number of cases/persons included in the analysis. For the sake of clarity, only significant results (p < 0.05) are shown, but the R² relates to the complete models with all included independent variables. (black font = significant influence). ^a^ multiple linear regression (mlr) with the health indicator Physical impairments as the outcome (dependent variable) and all work related potentials and risks as exposition (20 independent variables). The criteria for the mlr were all met. ^b^ like ^a^ but additionally adjusted for age, sex, and branch of industry.

Supplementary Table 8: A table to show the multiple linear regression with Physical impairments as the outcome for poor sleepers

| **Outcome: Physical impairments** | **Little/No Sleeping Problems** | | | | | | | | |
| --- | --- | --- | --- | --- | --- | --- | --- | --- | --- |
|  | **Model 1^a^** | | R²: 24.4% N = 5,915 | | **Model 2^b^** | | R²: 27.5% N = 4,667 | | |
|  | β | 95%CI | | p | β | 95%CI | | p |  |
|  |  | low | high |  |  | low | high |  |  |
| **Identification** *(i.e. being convinced of the company's products/services; company has a future)* | -0,033 | -0,050 | -0,016 | <0,001 | -0,032 | -0,052 | -0,012 | 0,001 |  |
| **Learning at work** *(i.e. developing skills & abilities)* | -0,055 | -0,070 | -0,040 | <0,001 | -0,052 | -0,069 | -0,035 | <0,001 |  |
| **Ergonomic-environmental stress** *(i.e. unsuitable seating/office chairs)* | 0,051 | 0,036 | 0,065 | <0,001 | 0,061 | 0,045 | 0,078 | <0,001 |  |
| **Physical-environmental stress** *(i.e. exposure to dust, drafts)* | 0,115 | 0,097 | 0,133 | <0,001 | 0,123 | 0,103 | 0,142 | <0,001 |  |
| **Technical overload** *(i.e. not adequately prepared for the tasks)* | 0,074 | 0,055 | 0,092 | <0,001 | 0,085 | 0,065 | 0,104 | <0,001 |  |
| **Physical strain** *(i.e. working in a constrained posture)* | 0,080 | 0,065 | 0,095 | <0,001 | 0,067 | 0,050 | 0,084 | <0,001 |  |
| **Fair assessment** *(i.e. superior does not favor certain colleagues)* | -0,036 | -0,056 | -0,016 | <0,001 | -0,036 | -0,058 | -0,014 | 0,002 |  |
| **Job insecurity** *(i.e. taking on a lesser job within the company)* | 0,034 | 0,020 | 0,049 | <0,001 | 0,038 | 0,022 | 0,053 | <0,001 |  |
| **Technical exchange with colleagues** *(i.e. exchange between colleagues is possible)* | -0,031 | -0,045 | -0,017 | <0,001 | -0,025 | -0,041 | -0,009 | 0,002 |  |
| **Technical support from leadership** *(i.e. superior can answer technical questions comprehensibly)* | 0,039 | 0,018 | 0,059 | <0,001 | 0,031 | 0,009 | 0,054 | 0,007 |  |
| **Time pressure** *(i.e. not enough time to carefully plan the tasks)* | 0,043 | 0,029 | 0,057 | <0,001 | 0,033 | 0,018 | 0,049 | <0,001 |  |
| **Leadership** *(i.e. superior makes clear and comprehensible decisions in difficult situations)* | -0,027 | -0,051 | -0,003 | 0,030 | -0,013 | -0,040 | 0,014 | 0,334 |  |
| **Information & participation in the company** | -0,023 | -0,042 | -0,004 | 0,018 | -0,017 | -0,038 | 0,004 | 0,119 |  |
| **Scope of decision making** | 0,016 | 0,002 | 0,030 | 0,021 | 0,015 | -0,001 | 0,031 | 0,059 |  |

β: non standardized regression coefficient; 95%CI: confidence interval; p: p-value; R²: adjusted explained variation; N = number of cases/persons included in the analysis. For the sake of clarity, only significant results (p < 0.05) are shown, but the R² relates to the complete models with all included independent variables. (black font = significant influence). ^a^ multiple linear regression (mlr) with the health indicator Physical impairments as the outcome (dependent variable) and all work related potentials and risks as exposition (20 independent variables). The criteria for the mlr were all met. ^b^ like ^a^ but additionally adjusted for age, sex, and branch of industry.

Supplementary Table 9: A table to show the multiple linear regression with Physical impairments as the outcome for good sleepers

| **Outcome: Psychological impairments** | **Moderate/Severe Sleeping Problems** | | | | | | | |
| --- | --- | --- | --- | --- | --- | --- | --- | --- |
|  | **Model 1^a^** | | R²: 31.0% N = 2,985 | | **Model 2^b^** | | R²: 30.9% N = 2,461 | |
|  | β | 95%CI | | p | β | 95%CI | | p |
|  |  | low | high |  |  | low | high |  |
| **Identification** *(i.e. being convinced of the company's products/services; company has a future)* | -0,072 | -0,100 | -0,044 | <0,001 | -0,062 | -0,095 | -0,029 | <0,001 |
| **Scope of decision making** *(i.e. organizing work as one sees fit)* | 0,032 | 0,008 | 0,057 | 0,010 | 0,029 | 0,001 | 0,056 | 0,043 |
| **Technical overload** *(i.e. not adequately prepared for the tasks)* | 0,132 | 0,102 | 0,161 | <0,001 | 0,132 | 0,099 | 0,165 | <0,001 |
| **Information & participation in the company** *(i.e. adequately informed about upcoming changes and decisions)* | -0,043 | -0,080 | -0,006 | 0,023 | -0,055 | -0,097 | -0,013 | 0,010 |
| **Learning at work** *(i.e. not losing skills/abilities)* | -0,078 | -0,104 | -0,051 | <0,001 | -0,084 | -0,115 | -0,054 | <0,001 |
| **Time pressure** *(i.e. not enough time to finish the tasks)* | 0,146 | 0,121 | 0,172 | <0,001 | 0,137 | 0,108 | 0,166 | <0,001 |
| **Interruptions** *(i.e. due to missing information/documents)* | 0,063 | 0,032 | 0,094 | <0,001 | 0,070 | 0,035 | 0,105 | <0,001 |
| **Job insecurity** *(i.e. taking on a lesser job within the company)* | 0,046 | 0,024 | 0,069 | <0,001 | 0,054 | 0,029 | 0,079 | <0,001 |
| **Physical-environmental stress** *(i.e. spatial confinement)* | 0,084 | 0,055 | 0,114 | <0,001 | 0,090 | 0,057 | 0,123 | <0,001 |
| **Ergonomic-environmental stress** *(i.e. unsuitable software)* | 0,035 | 0,012 | 0,058 | 0,003 | 0,021 | -0,006 | 0,047 | 0,121 |
| **Information & participation at the workplace** *(i.e. personal suggestions are taken into account)* | 0,064 | 0,024 | 0,105 | 0,002 | 0,070 | 0,025 | 0,114 | 0,002 |
| **Work organization** *(i.e. work processes are well organized)* | -0,049 | -0,091 | -0,007 | 0,021 | -0,054 | -0,100 | -0,008 | 0,021 |
| **Working climate** *(i.e. addressing difficulties or problems without becoming unpopular)* | -0,051 | -0,078 | -0,025 | <0,001 | -0,047 | -0,077 | -0,017 | 0,002 |

β: non standardized regression coefficient; 95%CI: confidence interval; p: p-value; R²: adjusted explained variation; N = number of cases/persons included in the analysis. For the sake of clarity, only significant results (p < 0.05) are shown, but the R² relates to the complete models with all included independent variables. (black font = significant influence). ^a^ multiple linear regression (mlr) with the health indicator Psychological impairments as the outcome (dependent variable) and all work related potentials and risks as exposition (20 independent variables). The criteria for the mlr were all met. ^b^ like ^a^ but additionally adjusted for age, sex, and branch of industry.

Supplementary Table 10: A table to show the multiple linear regression with Psychological impairments as the outcome for poor sleepers

| **Outcome: Psychological impairments** | **Little/No Sleeping Problems** | | | | | | | |
| --- | --- | --- | --- | --- | --- | --- | --- | --- |
|  | **Model 1^a^** | | R²: 31.8% N = 5,915 | | **Model 2^b^** | | R²: 32.8% N = 4,667 | |
|  | β | 95%CI | | p | β | 95%CI | | p |
|  |  | low | high |  |  | low | high |  |
| **Identification** *(i.e. being convinced of the company's products/services; company has a future)* | -0,068 | -0,087 | -0,049 | <0,001 | -0,063 | -0,085 | -0,041 | <0,001 |
| **Scope of decision making** *(i.e. organizing work as one sees fit)* | 0,028 | 0,013 | 0,043 | <0,001 | 0,031 | 0,014 | 0,049 | <0,001 |
| **Technical overload** *(i.e. not adequately prepared for the tasks)* | 0,135 | 0,115 | 0,155 | <0,001 | 0,144 | 0,122 | 0,167 | <0,001 |
| **Information & participation in the company** *(i.e. adequately informed about upcoming changes and decisions)* | -0,024 | -0,045 | -0,003 | 0,022 | -0,026 | -0,050 | -0,003 | 0,029 |
| **Learning at work** *(i.e. not losing skills/abilities)* | -0,073 | -0,090 | -0,057 | <0,001 | -0,077 | -0,097 | -0,058 | <0,001 |
| **Time pressure** *(i.e. not enough time to finish the tasks)* | 0,125 | 0,109 | 0,140 | <0,001 | 0,116 | 0,099 | 0,133 | <0,001 |
| **Interruptions** *(i.e. due to missing information/documents)* | 0,062 | 0,042 | 0,082 | <0,001 | 0,057 | 0,034 | 0,079 | <0,001 |
| **Job insecurity** *(i.e. taking on a lesser job within the company)* | 0,026 | 0,011 | 0,042 | 0,001 | 0,032 | 0,015 | 0,049 | <0,001 |
| **Physical-environmental stress** *(i.e. spatial confinement)* | 0,053 | 0,033 | 0,072 | <0,001 | 0,054 | 0,032 | 0,076 | <0,001 |
| Fair assessment *(i.e. fair performance appraisal)* | -0,039 | -0,061 | -0,017 | 0,001 | -0,043 | -0,068 | -0,018 | 0,001 |
| Leadership *(i.e. superior ensures a good working climate)* | -0,039 | -0,066 | -0,012 | 0,004 | -0,034 | -0,064 | -0,004 | 0,024 |
| Technical exchange with colleagues *(i.e. exchange between colleagues is possible)* | -0,029 | -0,045 | -0,014 | <0,001 | -0,023 | -0,041 | -0,006 | 0,010 |
| Ergonomic-environmental stress *(i.e. unsuitable software)* | 0,027 | 0,011 | 0,043 | 0,001 | 0,027 | 0,009 | 0,046 | 0,003 |
| Physical strain *(i.e. working in a constrained posture)* | 0,053 | 0,036 | 0,069 | <0,001 | 0,052 | 0,033 | 0,071 | <0,001 |
| Information & participation at the workplace *(i.e. personal suggestions are taken into account)* | 0,031 | 0,005 | 0,056 | 0,019 | 0,018 | -0,010 | 0,047 | 0,214 |
| Work organization *(i.e. work processes are well organized)* | -0,037 | -0,063 | -0,010 | 0,007 | -0,025 | -0,054 | 0,004 | 0,094 |
| Technical support from leadership | 0,023 | 0,001 | 0,046 | 0,044 | 0,019 | -0,007 | 0,044 | 0,153 |

β: non standardized regression coefficient; 95%CI: confidence interval; p: p-value; R²: adjusted explained variation; N = number of cases/persons included in the analysis. For the sake of clarity, only significant results (p < 0.05) are shown, but the R² relates to the complete models with all included independent variables. ^a^ multiple linear regression (mlr) with the health indicator Psychological impairments as the outcome (dependent variable) and all work related potentials and risks as exposition (20 independent variables). The criteria for the mlr were all met. ^b^ like ^a^ but additionally adjusted for age, sex, and branch of industry. Supplementary Table 11: A table to show the multiple linear regression with Psychological impairments as the outcome for good sleepers

| **Outcome: Joy of work** | **Moderate/Severe Sleeping Problems** | | | | | | | |
| --- | --- | --- | --- | --- | --- | --- | --- | --- |
|  | **Model 1^a^** | | R²: 32.2%  N = 2,982 | | **Model 2^b^** | | R²: 32.6% N = 2,459 | |
|  | β | 95%CI | | p | β | 95%CI | | p |
|  |  | low | high |  |  | low | high |  |
| **Identification** *(i.e. being convinced of the company's products/services)* | 0,221 | 0,185 | 0,257 | <0,001 | 0,257 | 0,215 | 0,299 | <0,001 |
| **Learning at work** *(i.e. developing skills & abilities)* | 0,293 | 0,259 | 0,327 | <0,001 | 0,257 | 0,219 | 0,295 | <0,001 |
| **Technical overload** *(i.e. not adequately prepared for the tasks)* | -0,067 | -0,105 | -0,029 | <0,001 | -0,067 | -0,109 | -0,026 | 0,002 |
| **Scope of decision making** *(i.e. making a lot of own decisions)* | 0,109 | 0,078 | 0,141 | <0,001 | 0,118 | 0,083 | 0,153 | <0,001 |
| **Development opportunities** *(i.e. good training opportunities)* | 0,072 | 0,037 | 0,106 | <0,001 | 0,055 | 0,017 | 0,093 | 0,004 |
| **Work organization** *(i.e. work processes are well organized)* | 0,082 | 0,028 | 0,135 | 0,003 | 0,090 | 0,032 | 0,148 | 0,002 |
| **Physical strain** *(i.e. working in a constrained posture)* | 0,082 | 0,050 | 0,114 | <0,001 | 0,087 | 0,052 | 0,123 | <0,001 |
| **Fair assessment** *(i.e. fair performance appraisal)* | -0,046 | -0,090 | -0,003 | 0,038 | -0,043 | -0,091 | 0,006 | 0,083 |
| **Interruptions** *(i.e. due to missing information/documents)* | 0,063 | 0,023 | 0,102 | 0,002 | 0,064 | 0,020 | 0,108 | 0,005 |

β: non standardized regression coefficient; 95%CI: confidence interval; p: p-value; R²: adjusted explained variation; N = number of cases/persons included in the analysis. For the sake of clarity, only significant results (p < 0.05) are shown, but the R² relates to the complete models with all included independent variables. ^a^ multiple linear regression (mlr) with the health indicator Joy of work as the outcome (dependent variable) and all work related potentials and risks as exposition (20 independent variables). The criteria for the mlr were all met.The criteria for the mlr were all met. ^b^ like ^a^ but additionally adjusted for age, sex, and branch of industry.

Supplementary Table 12: A table to show the multiple linear regression with Joy of work as the outcome for poor sleepers

|  |  |  |  |  |  |  |  |  |  |
| --- | --- | --- | --- | --- | --- | --- | --- | --- | --- |
| **Outcome: Joy of work** | **Little/No Sleeping Problems** | | | | | | | | |
|  | **Model 1^a^** | | R²: 37.7% N = 5,904 | | **Model 2^b^** | | R²: 37.9% N = 4,661 | | |
|  | β | 95%CI | | p | β | 95%CI | | p |  |
|  |  | low | high |  |  | low | high |  |  |
| **Identification** *(i.e. being convinced of the company's products/services)* | 0,232 | 0,207 | 0,257 | <0,001 | 0,251 | 0,222 | 0,280 | <0,001 |  |
| **Learning at work** *(i.e. developing skills & abilities)* | 0,299 | 0,277 | 0,320 | <0,001 | 0,298 | 0,273 | 0,323 | <0,001 |  |
| **Technical overload** *(i.e. not adequately prepared for the tasks)* | -0,085 | -0,111 | -0,059 | <0,001 | -0,095 | -0,124 | -0,066 | <0,001 |  |
| **Scope of decision making** *(i.e. making a lot of own decisions)* | 0,077 | 0,057 | 0,097 | <0,001 | 0,072 | 0,049 | 0,095 | <0,001 |  |
| **Development opportunities** *(i.e. good training opportunities)* | 0,042 | 0,021 | 0,063 | <0,001 | 0,051 | 0,027 | 0,074 | <0,001 |  |
| **Work organization** *(i.e. work processes are well organized)* | 0,041 | 0,006 | 0,075 | 0,022 | 0,041 | 0,003 | 0,079 | 0,035 |  |
| **Physical strain** *(i.e. working in a constrained posture)* | 0,019 | -0,003 | 0,040 | 0,091 | 0,038 | 0,013 | 0,063 | 0,003 |  |
| **Fair assessment** *(i.e. fair performance appraisal)* | -0,037 | -0,066 | -0,008 | 0,012 | -0,044 | -0,077 | -0,012 | 0,008 |  |
| **Technical support from leadership** | 0,032 | 0,002 | 0,061 | 0,035 | 0,034 | 0,000 | 0,068 | 0,047 |  |
| **Physical-environmental stress** | -0,034 | -0,060 | -0,008 | 0,010 | -0,022 | -0,051 | 0,007 | 0,134 |  |

β: non standardized regression coefficient; 95%CI: confidence interval; p: p-value; R²: adjusted explained variation; N = number of cases/persons included in the analysis. For the sake of clarity, only significant results (p < 0.05) are shown, but the R² relates to the complete models with all included independent variables. ^a^ multiple linear regression (mlr) with the health indicator Joy of work as the outcome (dependent variable) and all work related potentials and risks as exposition (20 independent variables). The criteria for the mlr were all met.The criteria for the mlr were all met. ^b^ like ^a^ but additionally adjusted for age, sex, and branch of industry.

Supplementary Table 13: A table to show the multiple linear regression with Joy of work as the outcome for good sleepers

| **Outcome: Confidence** | **Moderate/Severe Sleeping Problems** | | | | | | | |
| --- | --- | --- | --- | --- | --- | --- | --- | --- |
|  | **Model 1^a^** |  | R²: 13.2% N = 2,975 | | **Model 2^b^** | | R²: 14.5% N = 2,456 | |
|  | β | 95%CI | | p | β | 95%CI | | p |
|  |  | low | high |  |  | low | high |  |
| **Interruptions** *(i.e. due to missing or faulty material, information/documents)* | 0,049 | 0,017 | 0,081 | 0,003 | 0,041 | 0,006 | 0,077 | 0,022 |
| **Technical overload** *(i.e. tasks exceed technical skills)* | -0,200 | -0,231 | -0,170 | <0,001 | -0,204 | -0,237 | -0,170 | <0,001 |
| **Identification** (i.e. being convinced of the company's products/services) | 0,095 | 0,066 | 0,124 | <0,001 | 0,108 | 0,074 | 0,141 | <0,001 |
| **Job insecurity** *(i.e. potential job loss)* | -0,023 | -0,046 | 0,000 | 0,054 | -0,034 | -0,059 | -0,008 | 0,009 |
| **Scope of decision making** *(i.e. planning task independently; making a lot of own decisions)* | 0,114 | 0,088 | 0,139 | <0,001 | 0,127 | 0,099 | 0,156 | <0,001 |
| **Technical exchange with colleagues** *(i.e. exchange between colleagues is possible)* | 0,041 | 0,015 | 0,067 | 0,002 | 0,047 | 0,018 | 0,076 | 0,002 |
| **Information & participation in the company** *(i.e. personal initiative & commitment are required)* | -0,066 | -0,104 | -0,028 | 0,001 | -0,085 | -0,128 | -0,043 | <0,001 |
| **Fair assessment** *(i.e. fair performance appraisal)* | -0,041 | -0,076 | -0,006 | 0,022 | -0,049 | -0,088 | -0,010 | 0,015 |
| **Time pressure** *(i.e. not enough time to carefully plan the tasks)* | 0,039 | 0,013 | 0,066 | 0,003 | 0,040 | 0,010 | 0,069 | 0,008 |
| **Ergonomic-environmental stress** | -0,026 | -0,049 | -0,002 | 0,034 | -0,026 | -0,053 | 0,000 | 0,054 |

β: non standardized regression coefficient; 95%CI: confidence interval; p: p-value; R²: adjusted explained variation; N = number of cases/persons included in the analysis. For the sake of clarity, only significant results (p < 0.05) are shown, but the R² relates to the complete models with all included independent variables. ^a^ multiple linear regression (mlr) with the health indicator Confidence as the outcome (dependent variable) and all work related potentials and risks as exposition (20 independent variables). The criteria for the mlr were all met. ^b^ like ^a^ but additionally adjusted for age, sex, and branch of industry.

Supplementary Table 14: A table to show the multiple linear regression with Confidence as the outcome for poor sleepers

| **Outcome: Confidence** | **Little/No Sleeping Problems** | | | | | | | |
| --- | --- | --- | --- | --- | --- | --- | --- | --- |
|  | **Model 1^a^** | | R²: 19.2% N = 5,899 | | **Model 2^b^** | | R²: 21.2% N = 4,660 | |
|  | β | 95%CI | | p | β | 95%CI | | p |
|  |  | low | high |  |  | low | high |  |
| **Interruptions** *(i.e. due to missing or faulty material, information/documents)* | 0,042 | 0,021 | 0,064 | <0,001 | 0,042 | 0,018 | 0,066 | 0,001 |
| **Technical overload** *(i.e. tasks exceed technical skills)* | -0,226 | -0,248 | -0,205 | <0,001 | -0,216 | -0,240 | -0,193 | <0,001 |
| **Identification** (i.e. being convinced of the company's products/services) | 0,122 | 0,102 | 0,143 | <0,001 | 0,135 | 0,111 | 0,159 | <0,001 |
| **Job insecurity** *(i.e. potential job loss)* | -0,033 | -0,050 | -0,016 | <0,001 | -0,042 | -0,061 | -0,023 | <0,001 |
| **Scope of decision making** *(i.e. planning task independently; making a lot of own decisions)* | 0,088 | 0,071 | 0,104 | <0,001 | 0,085 | 0,066 | 0,104 | <0,001 |
| **Technical exchange with colleagues** *(i.e. exchange between colleagues is possible)* | 0,037 | 0,020 | 0,054 | <0,001 | 0,035 | 0,016 | 0,055 | <0,001 |
| **Information & participation in the company** *(i.e. personal initiative & commitment are required)* | -0,047 | -0,070 | -0,025 | <0,001 | -0,053 | -0,079 | -0,028 | <0,001 |
| **Fair assessment** *(i.e. fair performance appraisal)* | -0,027 | -0,051 | -0,003 | 0,027 | -0,027 | -0,054 | 0,000 | 0,047 |
| **Appreciation** *(i.e. standards of assessment are known)* | -0,025 | -0,046 | -0,004 | 0,018 | -0,024 | -0,047 | 0,000 | 0,047 |
| **Work organization** *(i.e. work processes are well organized)* | 0,065 | 0,036 | 0,094 | <0,001 | 0,061 | 0,029 | 0,092 | <0,001 |
| **Development opportunities** *(i.e. getting support with career opportunities)* | -0,031 | -0,048 | -0,013 | 0,001 | -0,021 | -0,040 | -0,002 | 0,033 |
| Information & participation at the workplace *(i.e. personal suggestions are taken into account)* | 0,042 | 0,014 | 0,069 | 0,003 | 0,055 | 0,024 | 0,086 | 0,001 |
| **Learning at work** *(i.e. developing skills & abilities)* | 0,059 | 0,040 | 0,077 | <0,001 | 0,055 | 0,034 | 0,075 | <0,001 |
| **Technical support from leadership** | 0,011 | -0,013 | 0,036 | 0,377 | 0,032 | 0,005 | 0,060 | 0,022 |
| **Ergonomic-environmental stress** | 0,007 | -0,010 | 0,025 | 0,408 | 0,021 | 0,001 | 0,041 | 0,038 |
| **Physical-environmental stress** | 0,031 | 0,010 | 0,053 | 0,004 | 0,022 | -0,002 | 0,045 | 0,075 |

β: non standardized regression coefficient; 95%CI: confidence interval; p: p-value; R²: adjusted explained variation; N = number of cases/persons included in the analysis. For the sake of clarity, only significant results (p < 0.05) are shown, but the R² relates to the complete models with all included independent variables. ^a^ multiple linear regression (mlr) with the health indicator Confidence as the outcome (dependent variable) and all work related potentials and risks as exposition (20 independent variables). The criteria for the mlr were all met. ^b^ like ^a^ but additionally adjusted for age, sex, and branch of industry. Supplementary Table 15: A table to show the multiple linear regression with Confidence as the outcome for good sleepers
